# Body Mass Index Is Associated with the Immunometabolic Profile in Psoriatic Arthritis: Real-Life Data

**DOI:** 10.3390/diseases14080296

**Published:** 2026-08-17

**Authors:** Christian D’Elia, Edda Russo, Giada Santagata, Viola Svelti, Serena Guiducci, Mariangela Manfredi, Maria Infantino, Francesca Li Gobbi, Maurizio Benucci

**Affiliations:** 1 Department of Experimental and Clinical Medicine, Division of Rheumatology, University of Florence, 50134 Florence, Italy; christian.delia@unifi.it (C.D.); giada.santagata@unifi.it (G.S.); viola.svelti@unifi.it (V.S.); serena.guiducci@unifi.it (S.G.); 2 Clinical Pathology S. Giuseppe Hospital, Azienda USL-Toscana Centro, 50053 Empoli, Italy; edda.russo@uslcentro.toscana.it; 3 Immunology and Allergology Laboratory Unit, S. Giovanni di Dio Hospital, Azienda USL-Toscana Centro, 50134 Florence, Italy; mariangela.manfredi@uslcentro.toscana.it (M.M.); maria2.infantino@uslcentro.toscana.it (M.I.); 4 Rheumatology Unit, S. Giovanni di Dio Hospital, Azienda USL-Toscana Centro, 50143 Florence, Italy; francesca.ligobbi@uslcentro.toscana.it

**Keywords:** body mass index, psoriatic arthritis, biomarkers, cardiovascular risk factors

## Abstract

**Background:** Psoriatic arthritis (PsA) is a chronic inflammatory disease frequently associated with obesity and metabolic comorbidities. Increasing evidence suggests that obesity is associated with systemic inflammation, insulin resistance, and adverse clinical outcomes; however, the immunometabolic profile associated with obesity in PsA has not been fully characterized. **Objective**: To investigate the relationship between obesity, inflammatory biomarkers, cytokine profiles, metabolic parameters, insulin resistance, and disease-related characteristics in patients with PsA. **Methods**: In this monocentric cross-sectional study, 224 consecutive patients fulfilling the Classification Criteria for Psoriatic Arthritis (CASPAR) for PsA were evaluated. Clinical, inflammatory, metabolic, and cytokine-related variables were analyzed within an integrated immunometabolic framework. Patients were stratified according to obesity status based on body mass index (BMI ≥ 30 kg/m^2^). Correlation analyses, multivariable regression analyses, and exploratory machine-learning approaches were applied to identify multidimensional patterns associated with obesity. Adjusted multivariable regression analyses were performed to account for available demographic, clinical, comorbidity-related, and therapeutic covariates. **Results**: Ninety-four patients (42.0%) were classified as obese and 130 (58.0%) as non-obese. Compared with non-obese patients, obese individuals exhibited significantly higher levels of C-reactive protein (CRP; 0.31 vs. 0.13 mg/dL, *p* < 0.001), erythrocyte sedimentation rate (ESR; 15.0 vs. 10.0 mm/h, *p* = 0.006), serum amyloid A (SAA; 7.7 vs. 6.4 mg/L, *p* = 0.008), insulin (8.0 vs. 6.1 μU/mL, *p* < 0.001), glycated hemoglobin (HbA1c; 38.0 vs. 37.0 mmol/mol, *p* = 0.003), Homeostasis Model Assessment of Insulin Resistance (HOMA-IR; 1.29 vs. 1.21, *p* = 0.007), triglycerides (106.0 vs. 85.0 mg/dL, *p* = 0.019), and Health Assessment Questionnaire (HAQ) scores (*p* < 0.001). Obese patients also showed a higher prevalence of hypertension (18.1% vs. 6.9%, *p* = 0.018). BMI was positively correlated with several inflammatory, metabolic, and disease-related variables, including CRP (ρ = 0.383), interleukin-6 (IL-6; ρ = 0.295), insulin (ρ = 0.289), ESR (ρ = 0.245), SAA (ρ = 0.228), HbA1c (ρ = 0.199), HAQ score (ρ = 0.351), and Disease Activity Index for Psoriatic Arthritis (DAPSA) score (ρ = 0.183), while showing an inverse correlation with high-density lipoprotein cholesterol (HDL-C) (ρ = −0.189). After adjustment for age, sex, disease duration, DAPSA, hypertension, type 2 diabetes mellitus, and current therapy class, obesity remained associated with higher CRP and fasting insulin levels and with greater odds of belonging to a higher HAQ category. Machine-learning analyses identified inflammatory biomarkers, insulin resistance indices, and lipid-related parameters as the most informative features associated with obesity-related phenotypes. **Conclusions**: Obesity in PsA was associated with a broader immunometabolic phenotype characterized by increased inflammatory burden, metabolic dysfunction, and worse functional outcomes. These findings support the integration of metabolic and cardiovascular assessment into routine rheumatology practice and highlight obesity as an important marker of increased immunometabolic and clinical burden in PsA. Although the cross-sectional design precludes causal inference, the observed associations may help identify patients requiring closer metabolic and cardiovascular assessment.

## 1. Introduction

**Figure 1:**
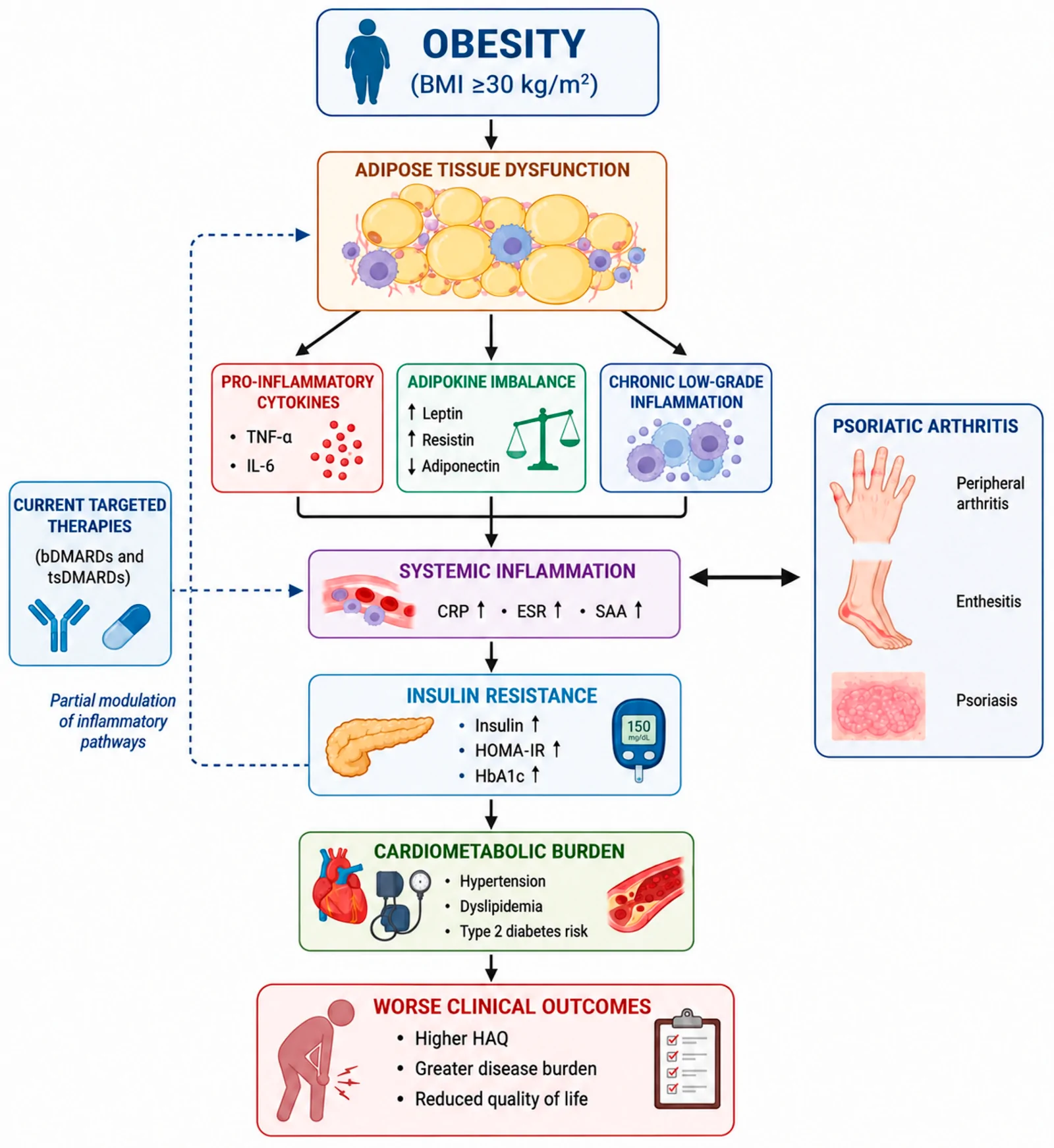
Conceptual framework of the immunometabolic phenotype associated with obesity in psoriatic arthritis. Graphical representation of the associations observed between obesity, adipose tissue dysfunction, systemic inflammation, insulin resistance, cardiometabolic burden, and clinical outcomes in patients with psoriatic arthritis. The diagram illustrates a conceptual framework integrating current evidence from the literature with the findings of the present study. Targeted therapies may modulate inflammatory pathways; however, obesity was associated with a broader immunometabolic phenotype characterized by increased inflammatory and metabolic burden. The figure was conceptualized by the authors and generated using artificial intelligence-assisted image generation tools, followed by author-directed scientific revision and editing.

Psoriatic Arthritis (PsA) is a chronic, immune-mediated inflammatory disease characterized by a complex, heterogeneous clinical presentation, encompassing peripheral arthritis, axial involvement, enthesitis, dactylitis, and psoriasis [1,2]. Beyond these classic musculoskeletal and cutaneous manifestations, PsA is increasingly recognized as a systemic condition [2,3]. This multifaceted nature is marked by a substantial burden of extra-articular comorbidities, which not only contribute to increased patient morbidity but also complicate clinical management and diminish long-term health outcomes [3,4]. Given the multisystemic involvement inherent to PsA, the recognition and assessment of these associated conditions have become essential pillars of modern rheumatological care [5]. Consequently, an integrated, multidisciplinary approach is increasingly required to effectively address the systemic inflammatory burden and optimize patient health outcomes, thereby ensuring comprehensive management of the disease [6]. Among the various comorbidities associated with PsA, obesity stands out as one of the most prevalent and clinically challenging [7,8]. Epidemiological evidence consistently indicates that the prevalence of obesity is markedly higher in individuals with PsA compared to the general population [2,8]. This elevated body mass is robustly associated with greater disease activity, increased functional impairment, and inferior patient-reported outcomes (PROs), fundamentally undermining the overall quality of life for affected individuals [8,9]. Furthermore, obesity significantly compromises therapeutic efficacy, as patients with higher body mass index (BMI) often demonstrate reduced responses to conventional and biologic therapies [10]. The mechanisms underlying these observations are likely multifactorial and involve a complex interplay between immune and metabolic pathways. Among the mechanisms implicated in these associations, adipose tissue has received considerable attention. In fact, adipose tissue functions as a metabolically active endocrine organ rather than a passive energy reservoir, actively secreting pro-inflammatory adipokines such as leptin and adiponectin that modulate innate and adaptive immune cell function [11,12]. In the context of obesity, this endocrine function becomes dysregulated, fostering a state of chronic, low-grade systemic inflammation [13,14]. Excessive adiposity facilitates the infiltration of pro-inflammatory immune cells, such as M1-polarized macrophages, into adipose tissue, triggering the release of key mediators including Tumor Necrosis Factor-alpha (TNF-α) and Interleukin-6 (IL-6) [15,16]. These inflammatory signals are not confined to the adipose compartment but significantly contribute to the systemic inflammatory burden [17]. The interplay between these circulating cytokines and metabolic regulation is linked to altered glucose homeostasis, often inducing peripheral insulin resistance [18]. Furthermore, this state of chronic immune activation appears to establish a self-amplifying feedback loop, where inflammatory pathways and metabolic dysfunction reciprocally reinforce one another [14]. Several of the cytokines implicated in obesity-associated inflammation are also central drivers of PsA pathogenesis. TNF-α, IL-6, and the IL-23/IL-17 axis represent key mediators of both local and systemic inflammatory responses in PsA [17,19]. Beyond their role in musculoskeletal disease, these cytokines have been linked to the modulation of metabolic pathways; specifically, elevated levels of TNF-α and IL-6 may contribute to the induction of insulin resistance and the dysregulation of adipokine secretion, establishing a mechanistic bridge between immune-mediated inflammation and metabolic dysfunction [11,15]. These cytokines represent key therapeutic targets in modern rheumatological care, with biologic (b-DMARDs) and targeted synthetic disease-modifying antirheumatic drugs (ts-DMARDs) designed to attenuate these specific signaling pathways [6,20]. Available evidence suggests that these interventions may influence systemic inflammatory biomarkers, circulating cytokine levels, and metabolic parameters, including glucose homeostasis and lipid profiles [20,21]. Consequently, in treated patient populations, the interpretation of immunometabolic profiles requires careful consideration of the modulatory effects exerted by ongoing pharmacological therapy [22,23]. Previous research has extensively documented the independent roles of obesity, systemic inflammatory burden, and metabolic dysfunction in patients with PsA [2,7,8]. However, these factors have predominantly been examined in isolation, leaving the complex, interconnected nature of their systemic influence insufficiently explored [11,14]. While available evidence suggests that comorbid conditions—such as dyslipidemia, insulin resistance, and heightened inflammatory markers—contribute to clinical heterogeneity and diminished therapeutic responses, the synergistic effect of these variables within a unified, multidimensional framework remains incompletely understood [3,4]. Furthermore, limited data are available regarding the concurrent evaluation of distinct therapeutic classes alongside a comprehensive panel of metabolic and cytokine parameters [20,23]. The immunometabolic phenotype of patients with PsA, particularly in the context of varying degrees of adiposity and ongoing treatment, has not been fully characterized (the conceptual framework underlying the present study is summarized in Figure 1).

The aim of this study was to investigate the relationship between obesity, inflammatory biomarkers, cytokine profiles, metabolic parameters, insulin resistance, and disease-related characteristics in patients with PsA. Clinical and laboratory variables were evaluated within an integrated immunometabolic framework. Exploratory machine-learning approaches were additionally applied to identify multidimensional patterns across the study population.

## 2. Materials and Methods

### 2.1. Study Design

This monocentric, observational, cross-sectional study was conducted at the Rheumatology Unit of S. Giovanni di Dio Hospital, Florence, Italy. Data were collected between December 2025 and May 2026. The study was designed to investigate the association between obesity, inflammatory biomarkers, metabolic parameters, insulin resistance, and disease-related characteristics in patients with PsA. The cross-sectional design allowed the assessment of clinical, laboratory, and metabolic variables at the time of data collection within a real-world rheumatology cohort.

### 2.2. Study Population

This study included consecutive adult patients (≥18 years) with PsA who attended the Rheumatology Unit of S. Giovanni di Dio Hospital, Florence, Italy, between December 2025 and May 2026. Eligibility required fulfillment of the Classification Criteria for Psoriatic Arthritis (CASPAR) [24]. Patients with incomplete clinical or laboratory data required for the predefined analyses were excluded. No additional exclusion criteria were applied.

### 2.3. Data Collection

Clinical and laboratory data were retrieved from the study database for each patient, encompassing demographic, clinical, metabolic, and therapeutic characteristics. Demographic and clinical variables included age, disease duration, Disease Activity in Psoriatic Arthritis (DAPSA), and Health Assessment Questionnaire (HAQ) scores. Inflammatory markers comprised erythrocyte sedimentation rate (ESR), C-reactive protein (CRP), serum amyloid A (SAA), and myeloid-related protein (MRP). Cytokine measurements included TNF-alpha, IL-6, IL-10, IL-17, and IL-8. Metabolic variables included fasting glucose, fasting insulin, glycated hemoglobin (HbA1c), and the homeostasis model assessment of insulin resistance (HOMA-IR). The lipid profile comprised total cholesterol, low-density lipoprotein (LDL) cholesterol, high-density lipoprotein (HDL) cholesterol, and triglycerides. Recorded comorbidities included hypertension, hypercholesterolemia, and type 2 diabetes mellitus (T2DM). Current treatments were also documented, including methotrexate, anti-TNF agents (adalimumab, etanercept, infliximab, and certolizumab pegol), anti-IL-17 therapies (secukinumab, ixekizumab, and bimekizumab), anti-IL-23 and IL-12/23-targeted therapies (guselkumab and ustekinumab), Janus kinase (JAK) inhibitors (tofacitinib and upadacitinib), and apremilast.

### 2.4. Laboratory Assessment

ESR was measured using an automated system (Alifax, Padova, Italy), and CRP levels were determined by immunoturbidimetric assay on a UniCel DxC 800 Synchron system (Beckman Coulter Inc., Brea, CA, USA). Fibrinogen levels (HemosIL QFA Thrombin, ACL TOP 750, Instrumentation Laboratory, Werfen, Bedford, MA, USA) and platelet count (Sysmex DI-60 system, Sysmex, Kobe, Japan) were assessed according to standard laboratory procedures. Serum cytokines, including IL-6 (Beckman Coulter Inc., Brea, CA, USA), IL-17 and TNF-alpha (R&D Systems, Minneapolis, MN, USA), IL-8 and IL-10 (eBiosci-ence/Bender MedSystem GmbH, Vienna, Austria), were measured using commercially available enzyme-linked immunosorbent assay (ELISA) kits. SAA was measured using an immunonephelometric method on a Nephelometer BNII (Siemens Healthcare Diagnostics, Marburg, Germany) and MRP levels were determined using ELISA immunoassay on Impatto twin Plus BRC (Eurospital, Trieste, Italy).

### 2.5. Obesity Definition

Body mass index (BMI) was calculated as weight in kilograms divided by the square of height in meters (kg/m^2^). According to the World Health Organization (WHO) classification criteria, obesity was defined as a BMI ≥ 30 kg/m^2^. Patients were subsequently categorized into obese and non-obese groups for comparative analyses.

### 2.6. HOMA-IR Calculation

Insulin resistance was estimated using the Homeostasis Model Assessment of Insulin Resistance index. HOMA-IR was calculated using the standard formula: fasting glucose (mg/dL) multiplied by fasting insulin (μU/mL), subsequently divided by a constant of 405 [25].

### 2.7. Ethics Statement

This study was based on the analysis of anonymized clinical and laboratory data collected during routine clinical practice, with no experimental intervention. The study was conducted within the framework of the GISEA Project and was approved by the relevant Ethics Committee on 22 September 2020 (approval code: 6496_OSS).

### 2.8. Statistical Analysis

Continuous variables were summarized as medians and interquartile ranges (IQRs) owing to the non-Gaussian distribution of most inflammatory and metabolic parameters. Comparisons between obese and non-obese patients were performed using the Mann–Whitney U test, whereas differences across multiple treatment groups were assessed using the Kruskal–Wallis test, with exploratory post hoc pairwise comparisons conducted when appropriate. Therapy-stratified analyses were restricted to patients receiving one of the predefined therapeutic categories (methotrexate, anti-TNF, anti-IL-17, anti-IL-23, JAK inhibitors, or apremilast). Patients with no recorded treatment (*n* = 9) or with unclassified treatment regimens (*n* = 1) were excluded from these analyses, resulting in a final sample of 214 patients. Categorical variables and comorbidity distributions were analyzed using Fisher’s exact test. To account for multiple statistical testing and reduce the risk of type I error, *p* values obtained from multiple comparisons were adjusted using the Benjamini–Hochberg false discovery rate (FDR) correction. 

To assess whether obesity was independently associated with the principal inflammatory, metabolic, and functional outcomes, additional multivariable regression analyses were performed. Obesity status (BMI ≥ 30 kg/m^2^) was included as the main explanatory variable, while age, sex, disease duration, disease activity assessed by DAPSA, hypertension, type 2 diabetes mellitus, and current therapy class were included as covariates selected a priori according to clinical relevance and data availability. Owing to their right-skewed distributions, CRP and fasting insulin were logarithmically transformed and analyzed using multivariable linear regression with heteroscedasticity-robust standard errors. Regression coefficients were back-transformed and reported as adjusted ratios with 95% confidence intervals. Because HAQ values represented ordered categories, HAQ was analyzed using ordinal logistic regression, with results reported as adjusted proportional odds ratios (OR) and 95% confidence intervals. The Benjamini–Hochberg false discovery rate correction was applied across the three principal adjusted outcome analyses. Information on cumulative corticosteroid exposure, lipid-lowering and glucose-lowering medications, previous PsA treatments, and treatment duration was not consistently available and could not be incorporated into the adjusted models.

An exploratory supervised machine-learning framework was implemented to investigate whether inflammatory, metabolic, and clinical variables could discriminate obesity-associated phenotypes within the psoriatic arthritis (PsA) cohort. To avoid information leakage, BMI was excluded from the predictive feature set. Three machine-learning algorithms were evaluated: Logistic Regression, Random Forest, and Gradient Boosting. Default hyperparameters implemented in the scikit-learn library were used for all models, as the primary objective of the machine-learning analyses was exploratory comparison rather than predictive model optimization. Therefore, no hyperparameter tuning or grid-search procedure was performed. Model performance is reported as the mean cross-validated area under the receiver operating characteristic curve (AUC) obtained across the five folds. Because the objective of the study was exploratory rather than predictive model development, confidence intervals were not estimated.

Missingness was minimal among the continuous clinical, inflammatory, and metabolic variables included in the machine-learning analysis. Three of 224 patients (1.34%) had at least one missing value, corresponding to 7 missing observations among 4704 evaluable data points (0.15%). Specifically, one value was missing for MRP, one for HOMA-IR, and one patient lacked measurements for TNF, IL-6, IL-10, IL-17, and IL-8. Binary comorbidity variables were coded as present or absent and were not treated as missing. To prevent information leakage, median imputation was fitted exclusively on each cross-validation training fold and subsequently applied to the corresponding validation fold. Standard scaling, when required by the specific algorithm, was likewise fitted within each training fold and then applied to the validation fold. Median imputation and standard scaling were applied independently within each training fold during the cross-validation procedure, and the corresponding transformations were subsequently applied to the validation fold, thereby preventing information leakage. Model performance was assessed using stratified 5-fold cross-validation to improve robustness and reduce overfitting. The use of repeated cross-validation was intended to reduce performance variability and provide a more robust estimate of model generalizability within the available dataset. The primary performance metric was the mean cross-validated area under the receiver operating characteristic curve (Mean CV AUC), while accuracy, balanced accuracy, sensitivity, and precision were evaluated as secondary metrics. Out-of-fold (OOF) predictions obtained during the cross-validation procedure were aggregated to generate receiver operating characteristic (ROC) curves, and the corresponding OOF AUC values were reported in the ROC plots. Default hyperparameters from the scikit-learn implementation were used because the primary objective was exploratory model comparison rather than algorithm optimization.

To enhance model interpretability, Random Forest feature importance was used as an exploratory measure to identify the inflammatory, metabolic, and clinical variables contributing most strongly to phenotype discrimination. Because impurity-based feature importance may be influenced by correlated predictors and variable type, these results should be interpreted cautiously and were not intended to represent a formal explainable artificial intelligence analysis.

Principal component analysis (PCA) was conducted after median imputation and z-score normalization of continuous variables to explore multidimensional inflammatory–metabolic patterns within the cohort, and explained variance ratios were calculated for each principal component. In addition, unsupervised k-means clustering was performed to investigate the presence of biologically distinct inflammatory–metabolic subgroups independently of predefined obesity or treatment categories. All analyses were performed using Python version 3.13 (Python Software Foundation, Wilmington, DE, USA), employing the pandas, NumPy, SciPy, scikit-learn, and matplotlib libraries. Statistical analyses, machine-learning modeling, dimensionality reduction, clustering procedures, and graphical visualizations were conducted within a unified computational environment to ensure analytical reproducibility.

## 3. Results

Metabolic–Inflammatory Phenotyping in Psoriatic Arthritis According to Obesity Status.

### 3.1. Study Population

A total of 224 patient records were identified in the study database and included in the final analysis. According to the predefined BMI threshold for obesity (≥30 kg/m^2^), 94 patients (42.0%) were classified as obese and 130 (58.0%) as non-obese (Table 1).

### 3.2. Comparison Between Obese and Non-Obese Patients

**Figure 2:**
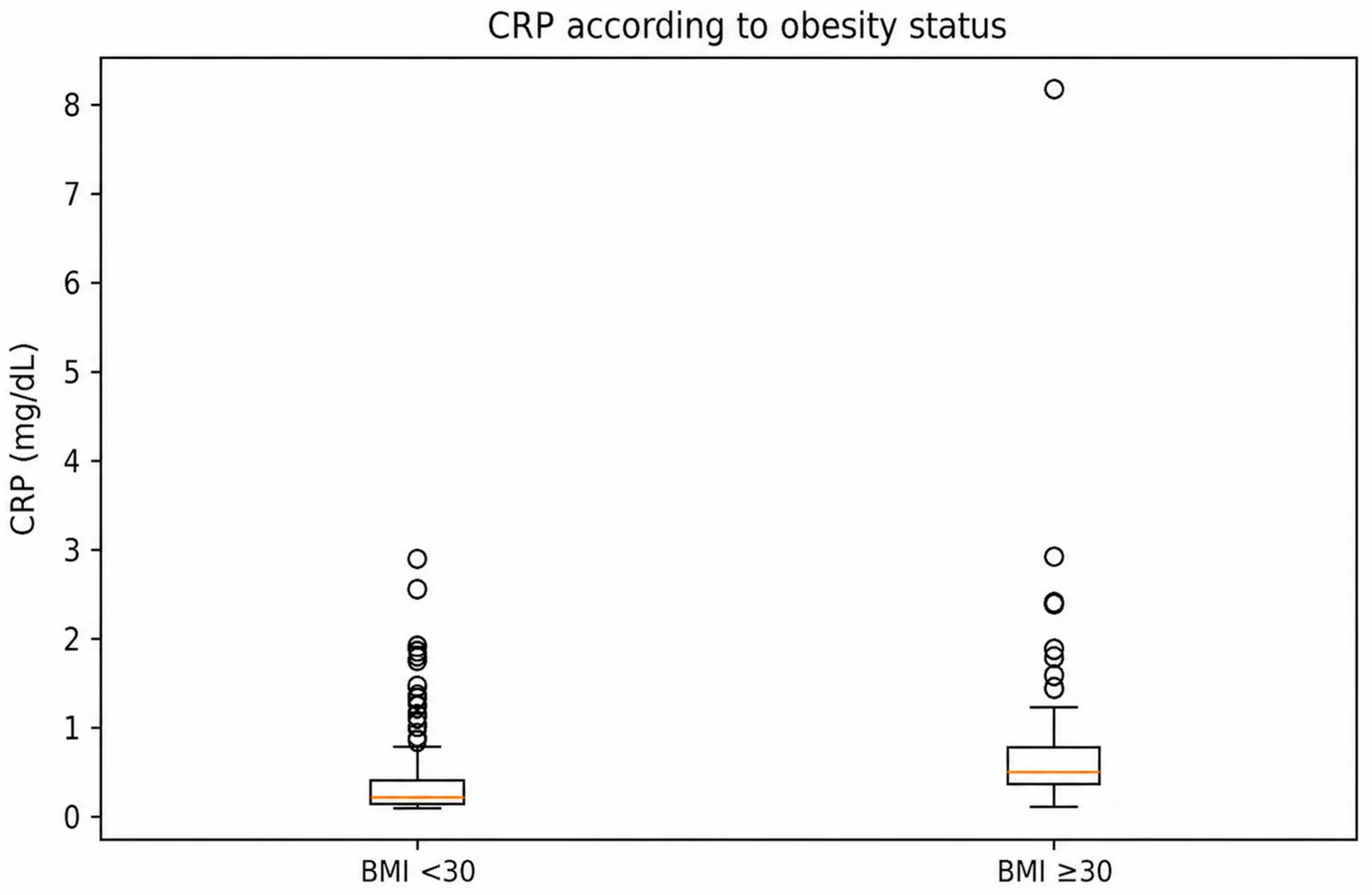
C-reactive protein (CRP) levels according to obesity status. Box-and-whisker plots showing the distribution of CRP levels (mg/dL) in patients with psoriatic arthritis stratified by obesity status (BMI < 30 kg/m^2^ and BMI ≥ 30 kg/m^2^). Boxes represent the interquartile range (IQR), the horizontal line indicates the median, whiskers extend to 1.5 × IQR, and circles represent outliers. Differences between groups were assessed using the Mann–Whitney U test.

**Figure 3:**
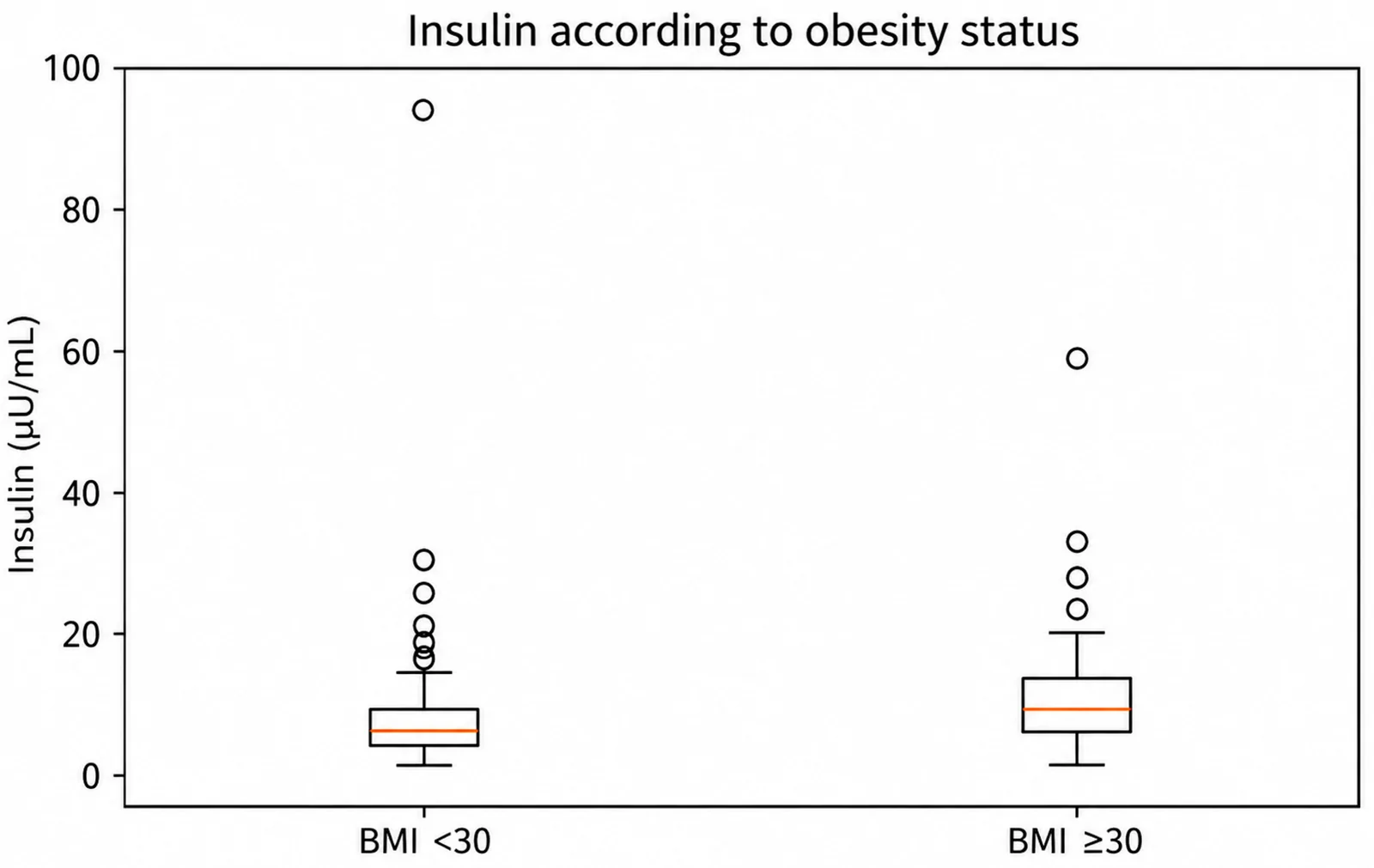
Insulin levels according to obesity status. Box-and-whisker plots showing the distribution of insulin levels (μU/mL) in patients with psoriatic arthritis stratified by obesity status (BMI < 30 kg/m^2^ and BMI ≥ 30 kg/m^2^). Boxes represent the interquartile range (IQR), the horizontal line indicates the median, whiskers extend to 1.5 × IQR, and circles represent outliers. Differences between groups were assessed using the Mann–Whitney U test.

**Figure 4:**
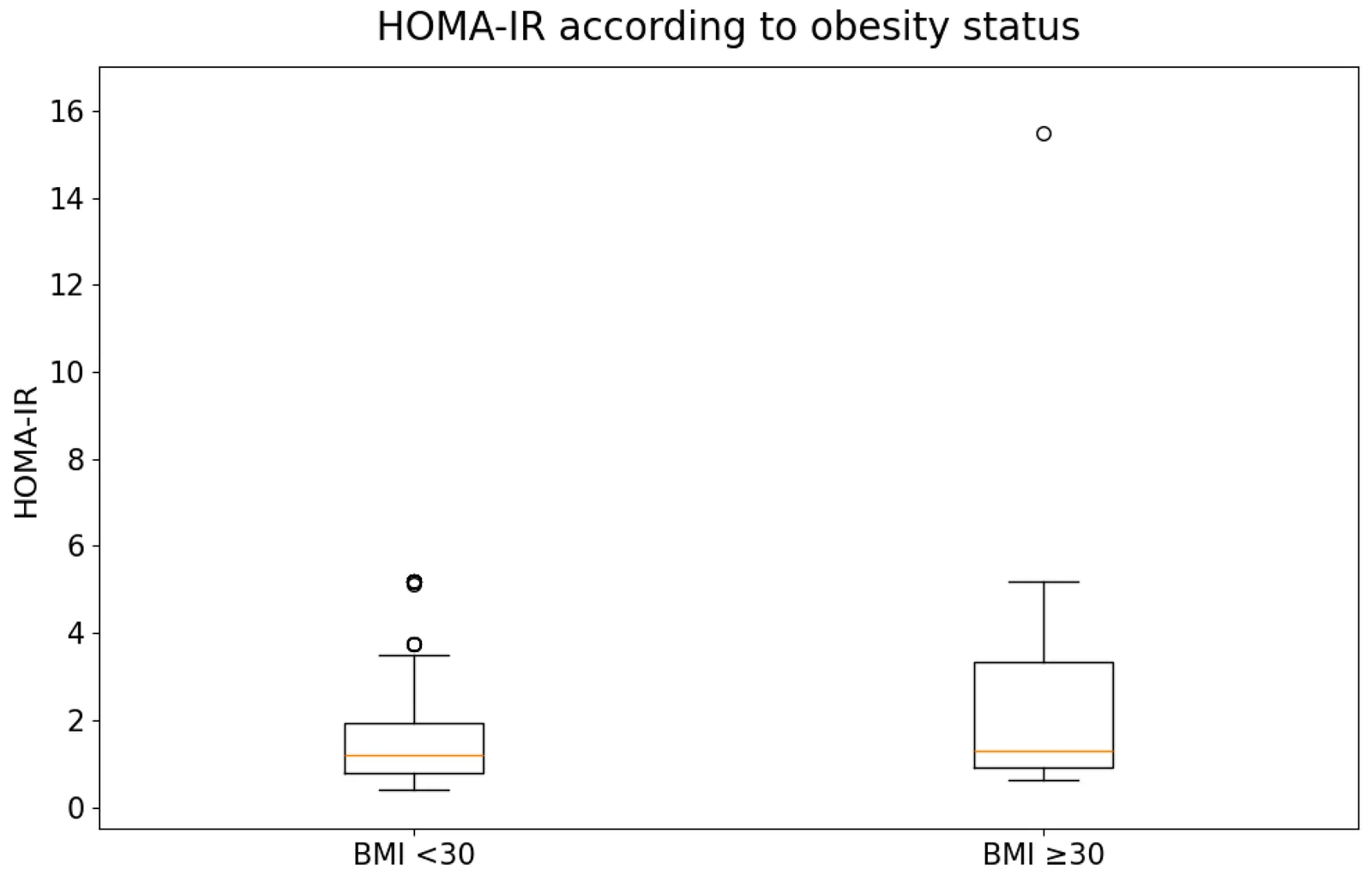
HOMA-IR according to obesity status. Box-and-whisker plots showing the distribution of the Homeostasis Model Assessment of Insulin Resistance (HOMA-IR) in patients with psoriatic arthritis stratified by obesity status (BMI < 30 kg/m^2^ and BMI ≥ 30 kg/m^2^). Boxes represent the interquartile range (IQR), the horizontal line indicates the median, whiskers extend to 1.5 × IQR, and circles represent outliers. Differences between groups were assessed using the Mann–Whitney U test.

BMI was significantly higher in obese patients (median 33.0 vs. 25.5 kg/m^2^, *p* < 0.001). Obese patients also exhibited higher levels of inflammatory markers, including CRP (0.31 vs. 0.13 mg/dL, *p* < 0.001), ESR (15.0 vs. 10.0 mm/h, *p* = 0.006), and SAA (7.7 vs. 6.4 mg/L, *p* = 0.008) (Figure 2). Metabolic alterations were likewise more pronounced among obese individuals, who showed higher fasting insulin levels (8.0 vs. 6.1 μU/mL, *p* < 0.001) (Figure 3), HbA1c values (38.0 vs. 37.0 mmol/mol, *p* = 0.003), HOMA-IR (1.29 vs. 1.21, *p* = 0.010) (Figure 4), and triglyceride concentrations (106.0 vs. 85.0 mg/dL, *p* = 0.019). In addition, obese patients had higher HAQ scores (0.50 vs. 0.50, *p* < 0.001), indicating greater functional impairment.

Several additional laboratory parameters, including TNF, IL-8, IL-10, total cholesterol, and LDL cholesterol, did not differ significantly between obese and non-obese patients after multiple-testing correction. Complete results for all measured laboratory variables are provided in Supplementary Table S2.

**Figure 5:**
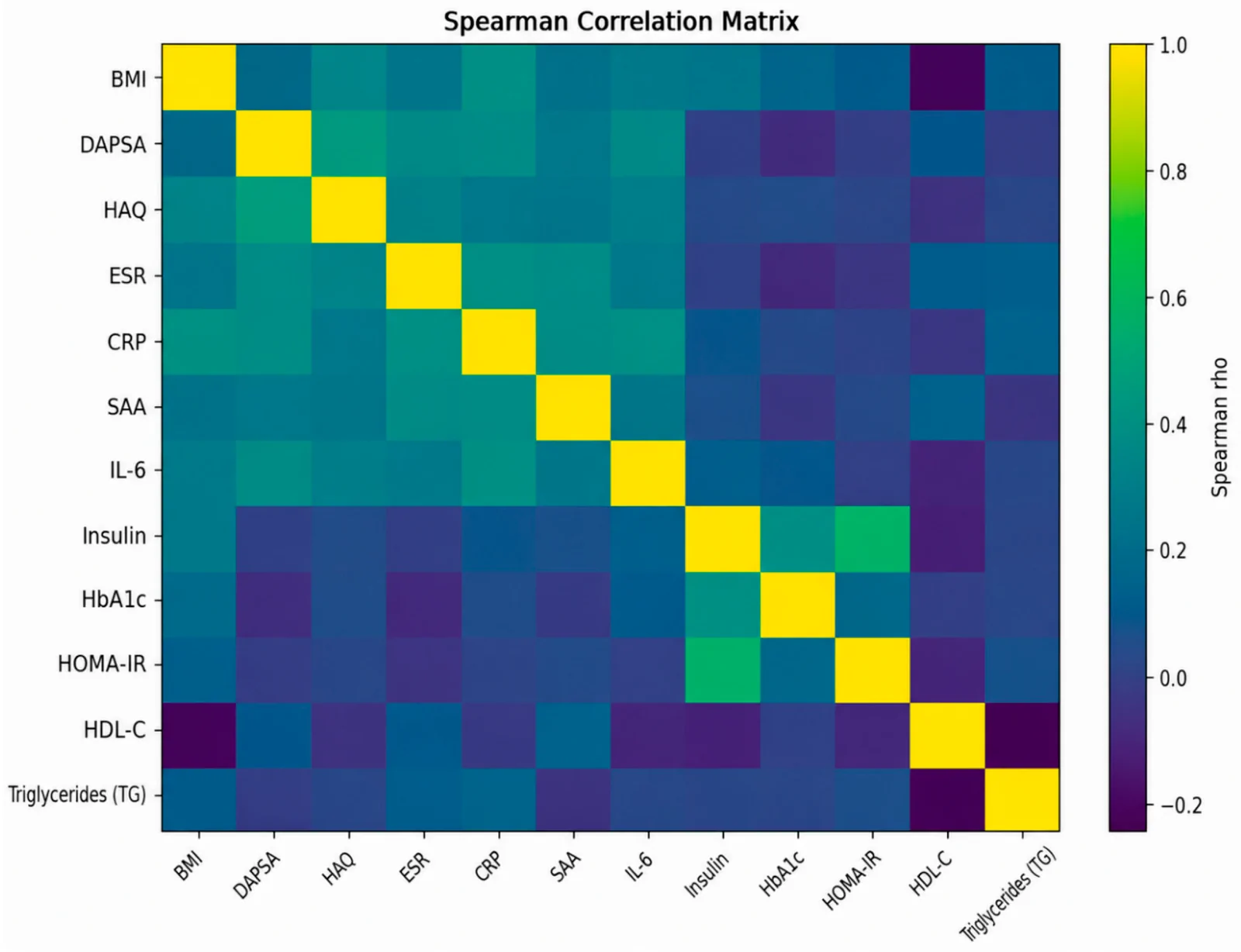
Correlation heatmap of inflammatory and metabolic variables.

Spearman correlation analyses identified a consistent association between increasing BMI and inflammatory-metabolic burden. Positive correlations were observed between BMI and CRP, ESR, SAA, IL-6, insulin, HOMA-IR, HbA1c, HAQ, and DAPSA. Conversely, HDL cholesterol showed a negative correlation trend with BMI. The overall correlation structure is illustrated in Figure 5.

In detail, Spearman correlation analysis revealed significant associations between BMI and multiple inflammatory, metabolic, and disease-related variables (Table 2). The strongest positive correlations were observed for CRP (ρ = 0.383, FDR-adjusted *p* < 0.0001) and HAQ score (ρ = 0.351, FDR-adjusted *p* < 0.0001), indicating that higher BMI values were associated with increased systemic inflammation and greater functional impairment. BMI was also positively correlated with IL-6 (ρ = 0.295, FDR-adjusted *p* = 0.0001) and insulin levels (ρ = 0.289, FDR-adjusted *p* = 0.0001), supporting a link between obesity, inflammatory activity, and insulin resistance. Additional significant positive correlations were observed with ESR (ρ = 0.245, FDR-adjusted *p* = 0.0009), SAA (ρ = 0.228, FDR-adjusted *p* = 0.0020), HbA1c (ρ = 0.199, FDR-adjusted *p* = 0.0084), and DAPSA score (ρ = 0.183, FDR-adjusted *p* = 0.0140). Conversely, HDL-C showed a significant inverse correlation with BMI (ρ = −0.189, FDR-adjusted *p* = 0.0119), indicating a less favorable lipid profile in patients with higher BMI values. BMI was also positively correlated with HOMA-IR.

### 3.3. Adjusted Multivariable Analyses

Multivariable analyses were performed to assess whether obesity contributes to the principal inflammatory, metabolic, and functional outcomes after adjustment for age, sex, disease duration, DAPSA, hypertension, type 2 diabetes mellitus, and current therapy class. Obesity was associated with higher CRP levels, corresponding to an adjusted 2.10-fold difference compared with non-obese patients (95% confidence interval [CI]: 1.55–2.85; *p* < 0.0001). Obesity was also associated with higher fasting insulin levels (adjusted ratio 1.32, 95% CI 1.11–1.56; *p* = 0.0018). In ordinal logistic regression, obese patients had greater odds of belonging to a higher HAQ category (adjusted proportional OR 3.10, 95% CI 1.79–5.37; *p* < 0.0001). All three associations remained statistically significant after false discovery rate correction (Table 3).

All models were adjusted for age, sex, disease duration, DAPSA, hypertension, type 2 diabetes mellitus, and current therapy class. Ratios for CRP and fasting insulin were obtained by exponentiating the regression coefficients from models using logarithmically transformed outcomes. A ratio greater than 1 indicates higher adjusted levels among obese patients.

## 4. Comorbidities

Among the evaluated comorbid conditions, hypertension was more prevalent among obese patients, supporting the concept that obesity in PsA is associated with a broader cardiometabolic phenotype (Table 4).

### 4.1. Machine-Learning Analysis

**Figure 6:**
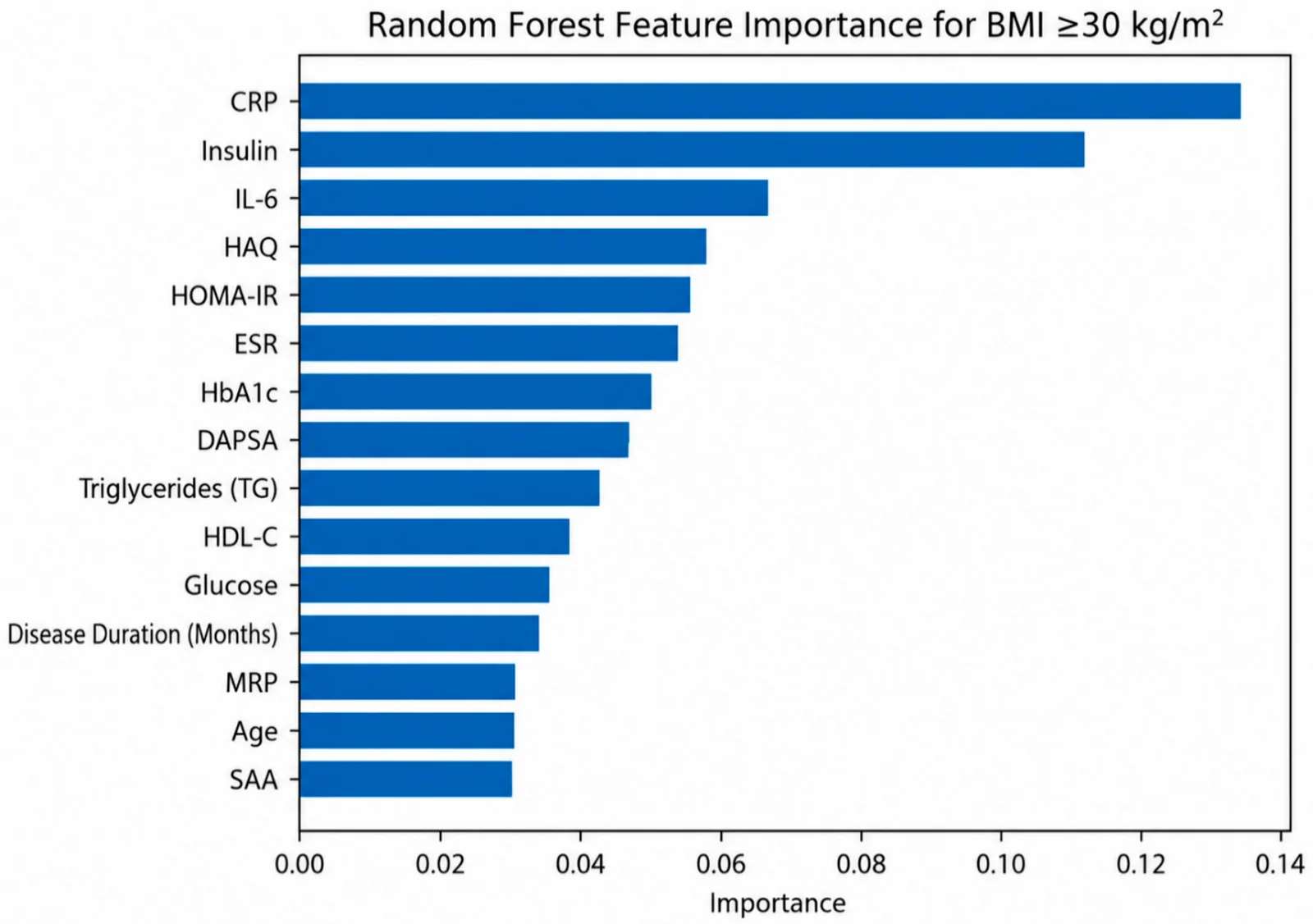
Random Forest feature importance analysis.

Supervised machine-learning models were subsequently applied to evaluate whether the combined clinical, inflammatory, and metabolic variables could discriminate obese from non-obese patients. Importantly, BMI itself was excluded from the predictive feature set in order to avoid information leakage. Overall, machine-learning analyses consistently identified inflammatory markers, insulin resistance indices, and lipid abnormalities among the most informative variables associated with obesity-related phenotypes. Feature-importance results are shown in Figure 6.

**Figure 7:**
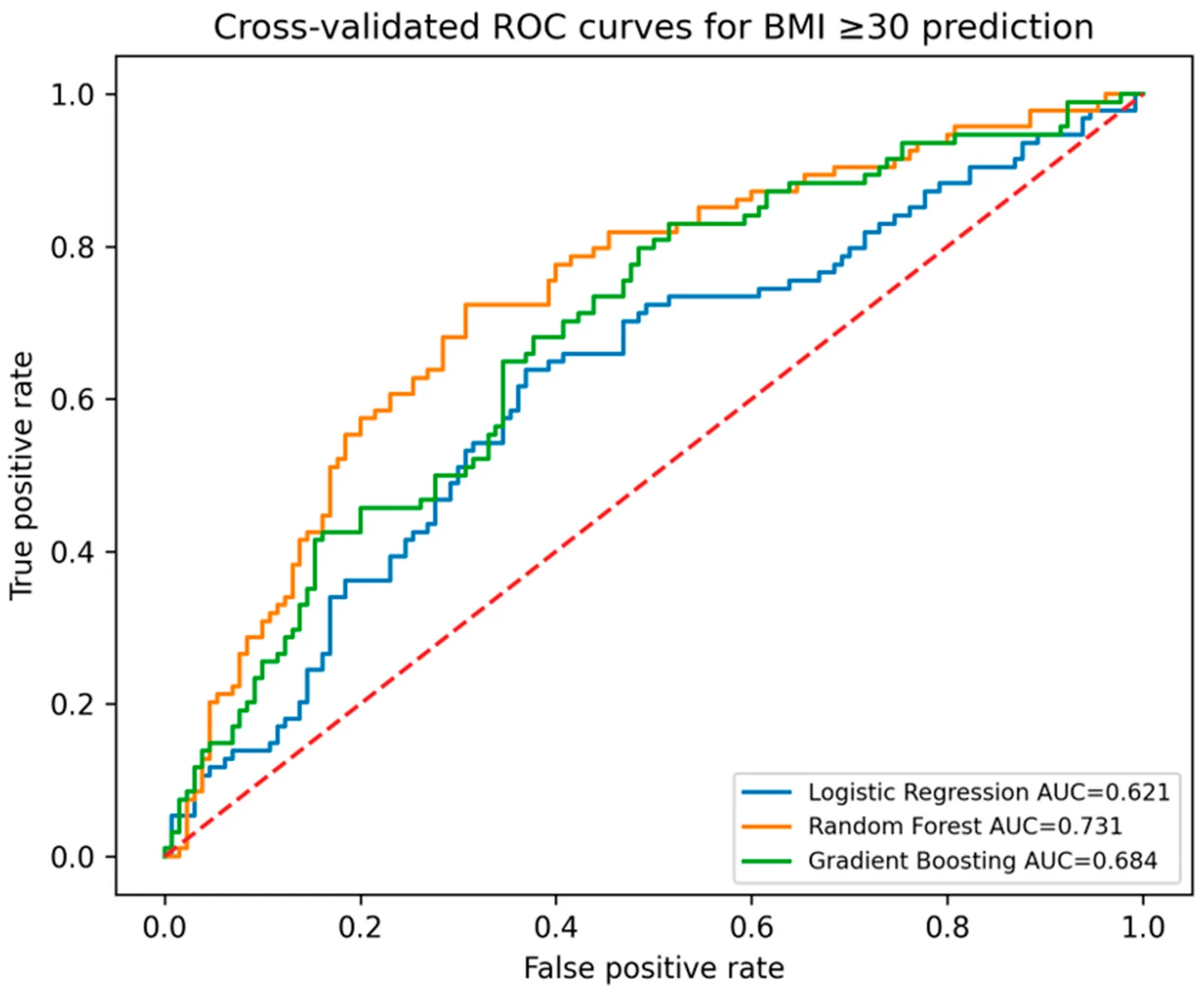
Receiver operating characteristic (ROC) curves of the supervised machine-learning models. ROC curves were generated from aggregated out-of-fold predictions obtained during stratified 5-fold cross-validation. The reported AUC values therefore correspond to the OOF AUC.

The best-performing model was Random Forest, which achieved a cross-validated AUC of 0.733. ROCs are shown in Figure 7. Detailed performance metrics are reported in Table 5.

Table 5 details the performance of the supervised machine-learning models for obesity prediction. Values represent the mean performance across the five cross-validation folds. The AUC values reported correspond to the mean cross-validated AUC (Mean CV AUC). ROC curves shown in Figure 7 were generated from out-of-fold (OOF) predictions; therefore, the corresponding OOF AUC values shown in the figure may differ slightly from the mean cross-validated AUC reported in this table.

### 4.2. Exploratory Feature Importance Analysis

Random Forest feature-importance analysis demonstrated that insulin resistance markers, acute-phase reactants, and lipid parameters contributed substantially to phenotype discrimination (Table 6).

### 4.3. Dimensionality Reduction and Clustering

**Figure 8:**
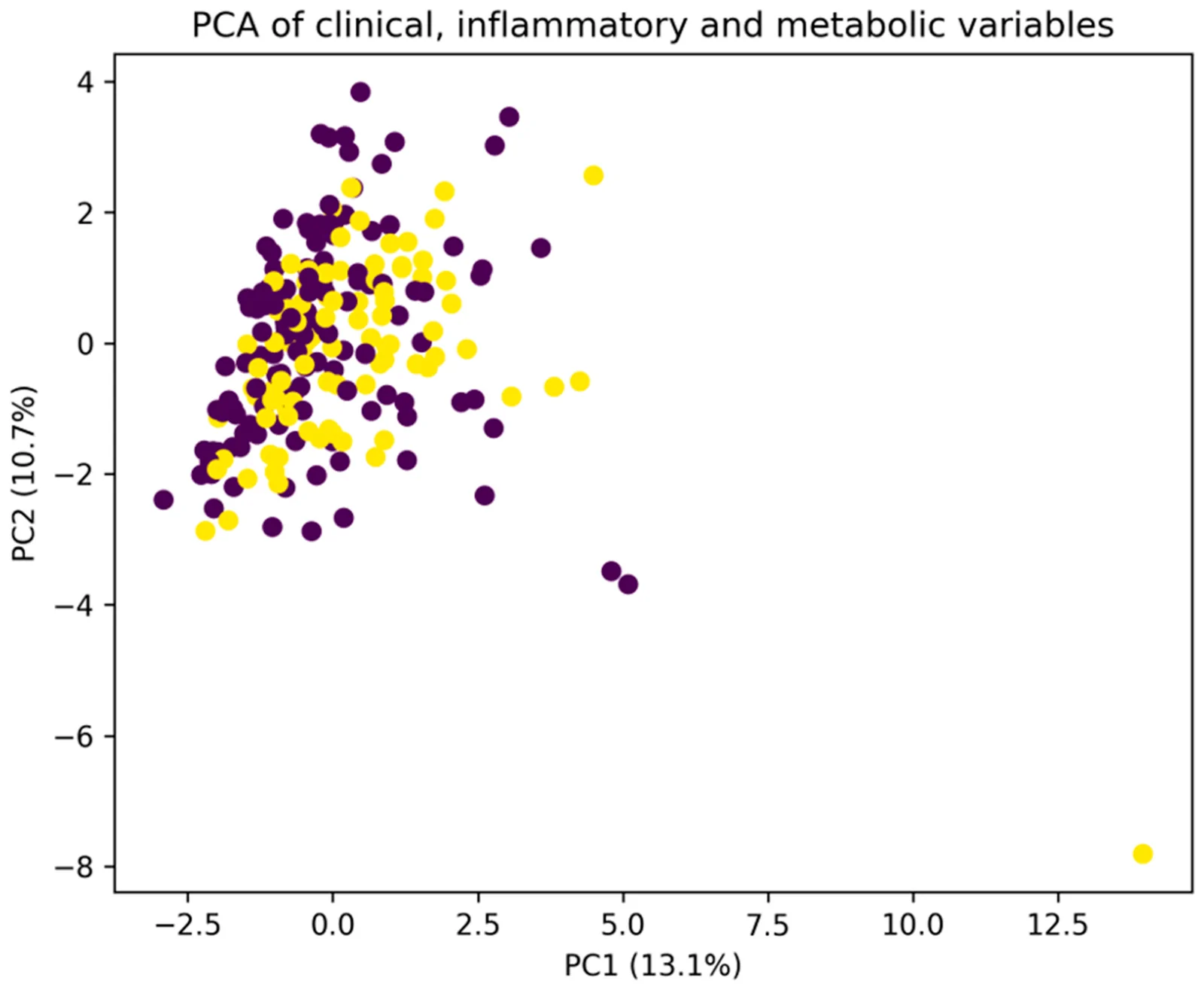
Principal component analysis (PCA) of clinical, inflammatory, and metabolic variables in patients with psoriatic arthritis. Each point represents one patient. Colors indicate the two clusters identified by the k-means clustering algorithm. The first two principal components explained 13.1% (PC1) and 10.7% (PC2) of the total variance.

Principal component analysis demonstrated partial separation between obese and non-obese patients within the multidimensional inflammatory-metabolic space. The first two principal components explained a clinically meaningful proportion of total variance, supporting the existence of structured inflammatory-metabolic phenotypes (Table 7). The PCA projection is shown in Figure 8.

### 4.4. Cluster Analysis Identified Two Major Phenotypic Groups

One cluster was characterized by higher BMI, increased inflammatory burden, greater insulin resistance, and higher prevalence of hypertension and diabetes-related variables, whereas the second cluster showed a comparatively milder metabolic-inflammatory profile.

### 4.5. Overall Interpretation

Taken together, these findings support the concept that obesity in PsA is not merely a coincidental comorbidity but rather part of a broader inflammatory-metabolic phenotype. The integration of inflammatory biomarkers, metabolic indices, functional assessment, and artificial intelligence (AI) approaches revealed multidimensional signatures associated with obesity-related disease burden. The predictive performance observed in the present study should be considered moderate and primarily hypothesis-generating. These models were intended to explore multidimensional relationships rather than to provide clinically deployable prediction tools.

### 4.6. Therapy-Stratified Inflammatory and Metabolic Profiling in Psoriatic Arthritis Study Population and Therapeutic Stratification

**Figure 9:**
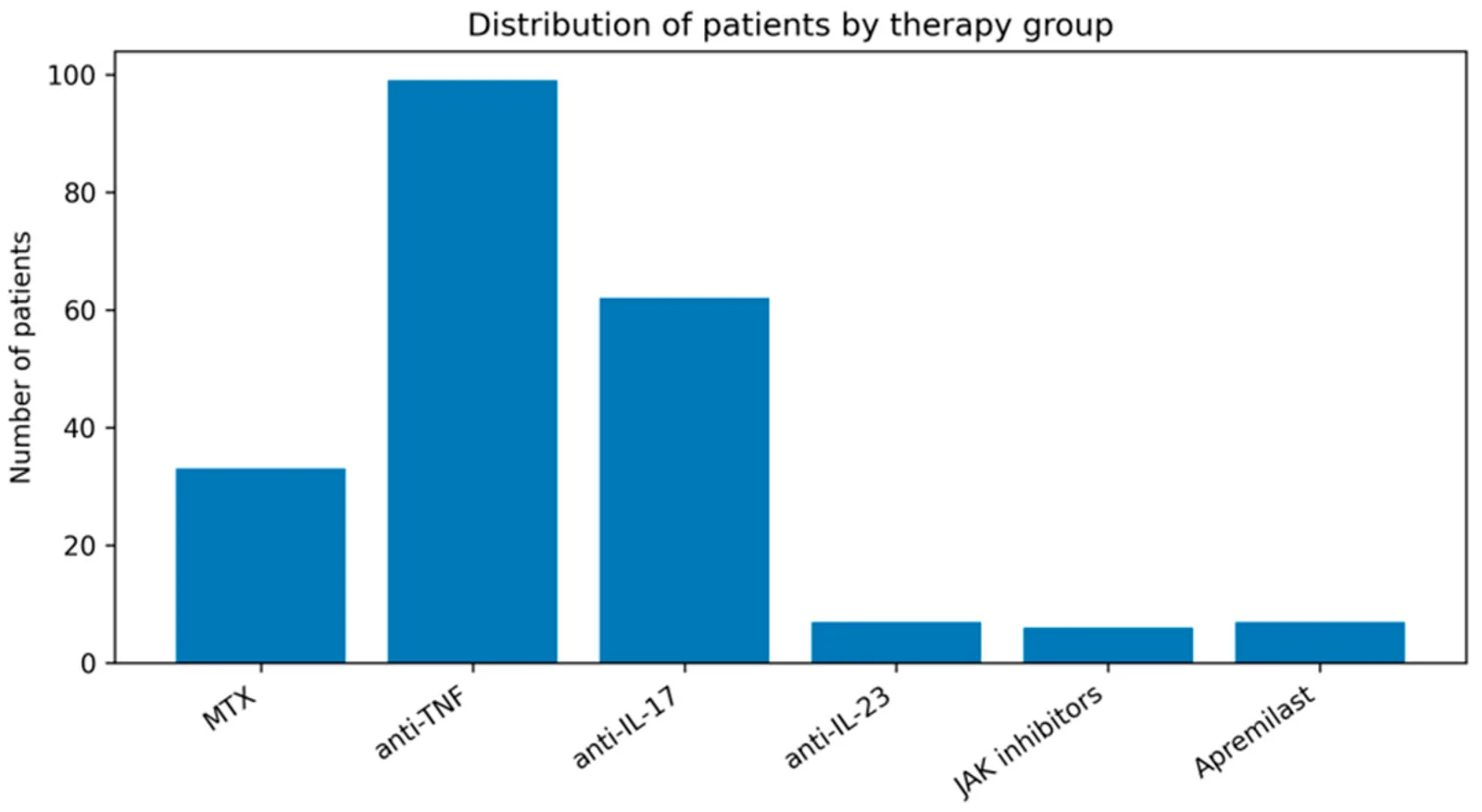
Distribution of patients according to therapeutic group. Patients were classified according to their current treatment as methotrexate (MTX), anti-TNF agents, anti-IL-17 therapies, anti-IL-23 therapies, JAK inhibitors, or apremilast. Therapy-stratified analyses included 214 patients with complete and classifiable treatment information; patients with no recorded treatment (*n* = 9) or unclassified treatment regimens (*n* = 1) were excluded.

A total of 224 patients with PsA were included in the therapy-stratified analysis. Patients were categorized according to the main therapeutic class into the following groups: methotrexate (MTX), anti-TNF agents (etanercept, infliximab, adalimumab), anti-IL-17 therapies (secukinumab, ixekizumab, bimekizumab), anti-IL-23 and IL-12/23 therapies (guselkumab and ustekinumab), JAK inhibitors (tofacitinib and upadacitinib), and apremilast (Table 8, Figure 9).

### 4.7. Comparative Analysis Across Therapeutic Groups

Comparative analyses demonstrated that the different therapeutic classes were associated with partially distinct inflammatory profiles, whereas metabolic and obesity-related variables showed a more heterogeneous distribution across treatment groups. Overall, the most evident differences involved inflammatory cytokines and acute-phase reactants. In particular, statistically significant differences across treatment groups were observed for TNF (Kruskal–Wallis *p* < 0.0001; FDR-adjusted *p* < 0.0001), IL-17 (*p* < 0.0001; FDR-adjusted *p* < 0.0001), SAA (*p* < 0.0001; FDR-adjusted *p* = 0.0001), and CRP (*p* = 0.0031; FDR-adjusted *p* = 0.0169).

**Figure 10:**
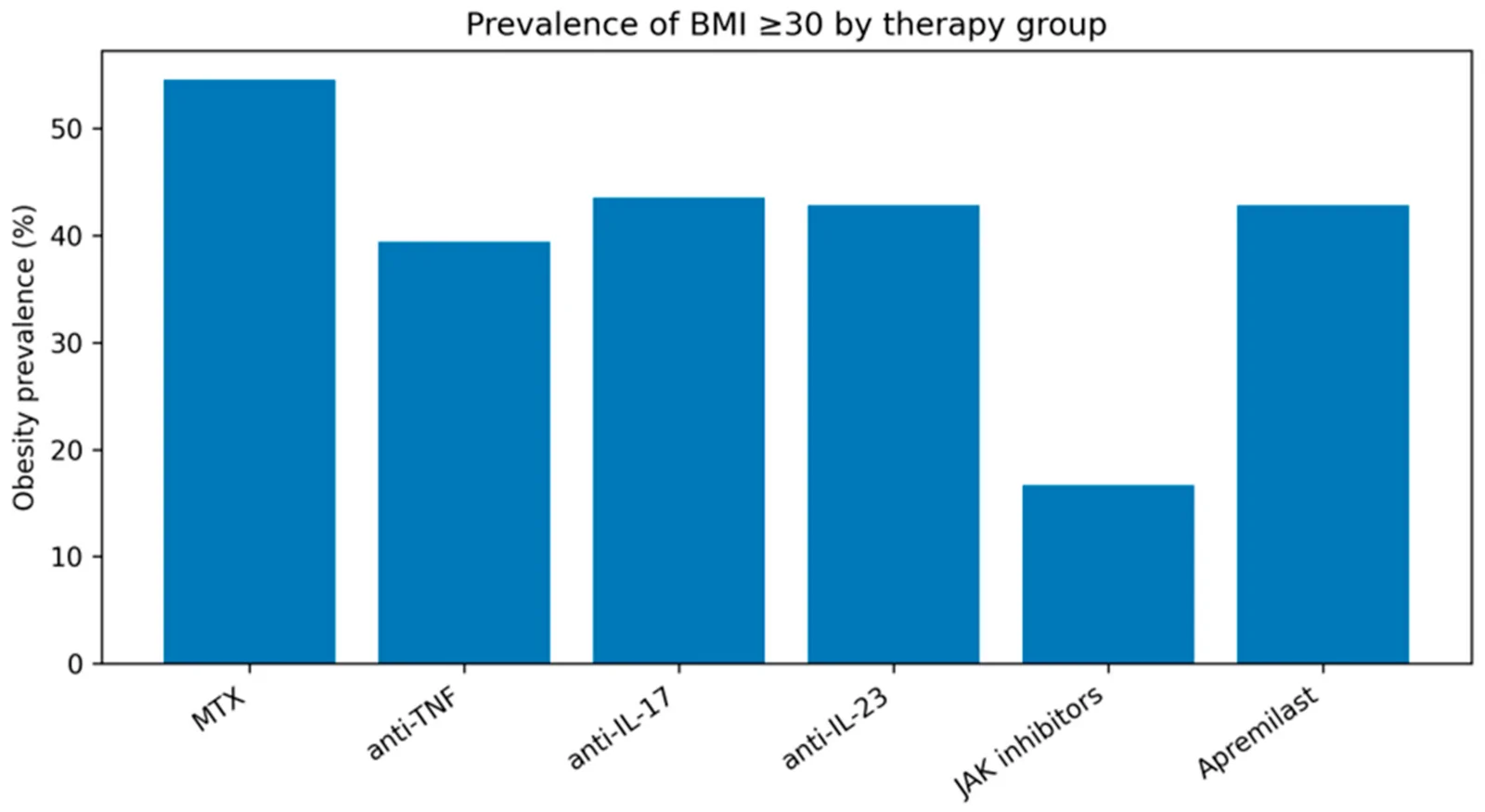
Prevalence of obesity according to therapeutic group. Bars represent the percentage of obese patients (BMI ≥ 30 kg/m^2^) within each therapeutic category. Therapy-stratified analyses included 214 patients with complete and classifiable treatment information; patients with no recorded treatment (*n* = 9) or unclassified treatment regimens (*n* = 1) were excluded.

Significant differences emerged for TNF-related inflammatory activity, IL-17-associated pathways, CRP, and SAA levels. Patients treated with anti-IL-17 therapies tended to show a distinct cytokine profile, whereas anti-TNF-treated patients demonstrated differences involving TNF-related inflammatory markers. At the same time, obesity prevalence and BMI distribution were relatively widespread across the different therapeutic classes (Figure 10), suggesting that metabolic burden may represent a transversal component of PsA independently of the specific biologic mechanism targeted.

### 4.8. Metabolic Variables and Obesity-Related Burden

**Figure 11:**
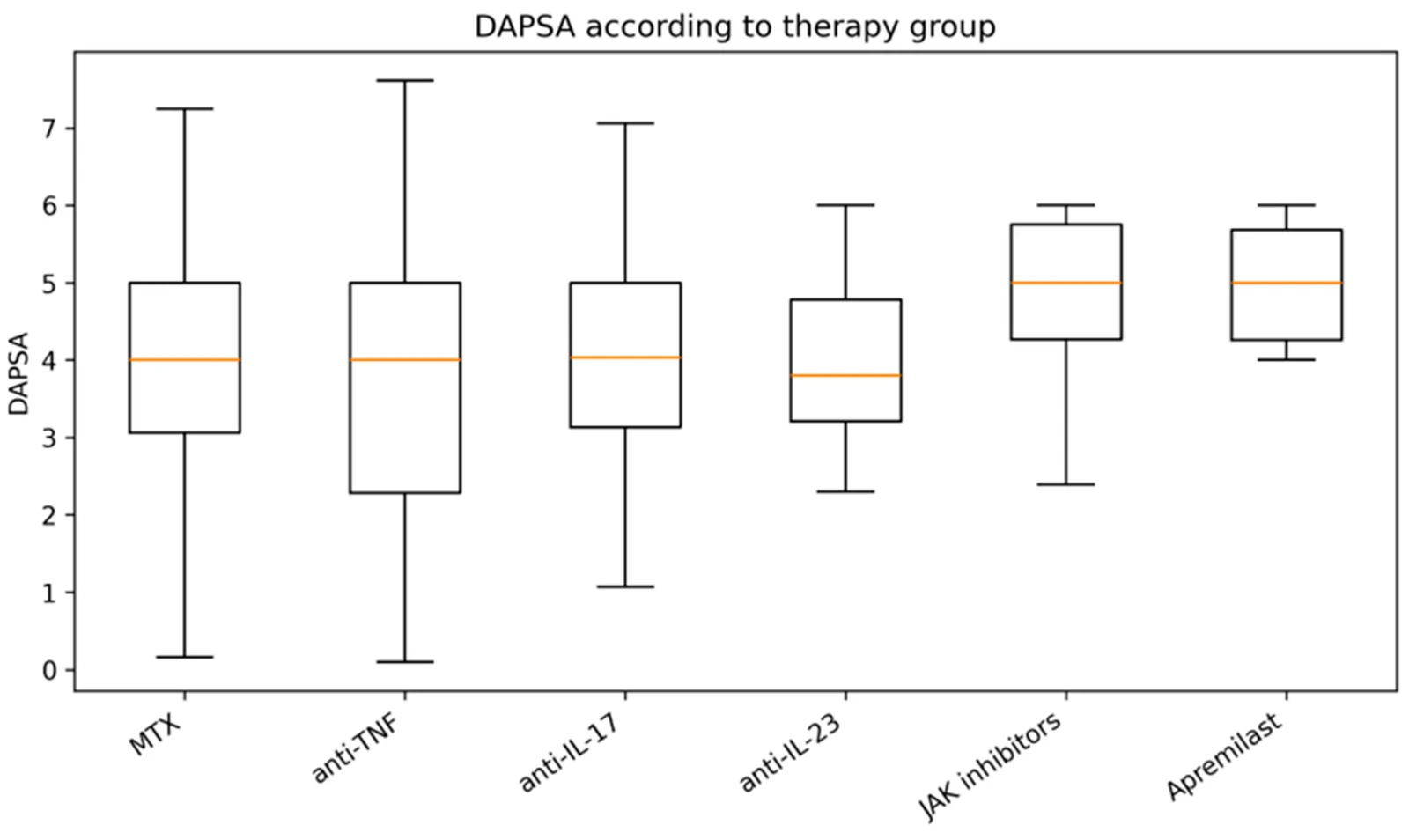
DAPSA according to therapeutic groups.

**Figure 12:**
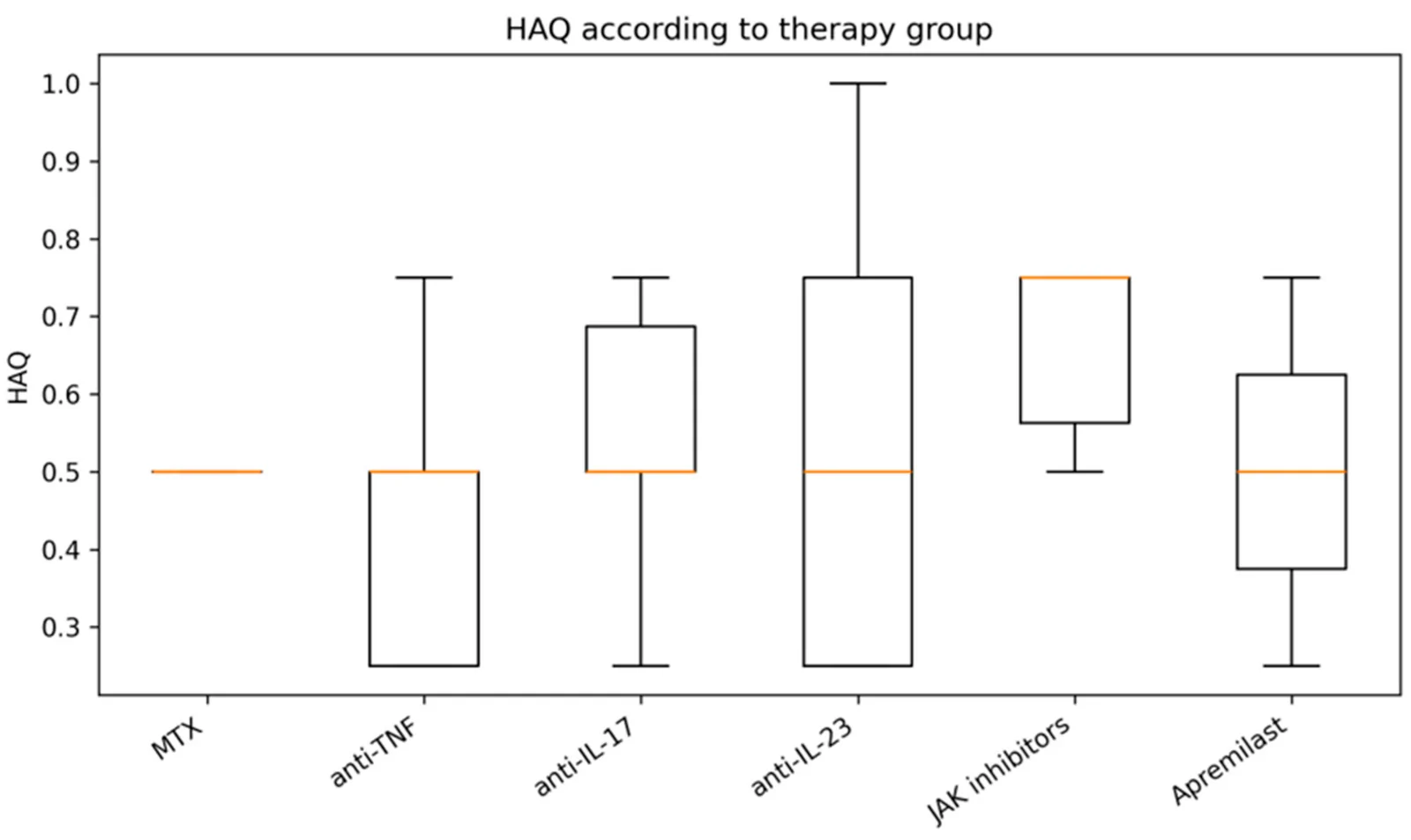
HAQ according to therapy stratification.

**Figure 13:**
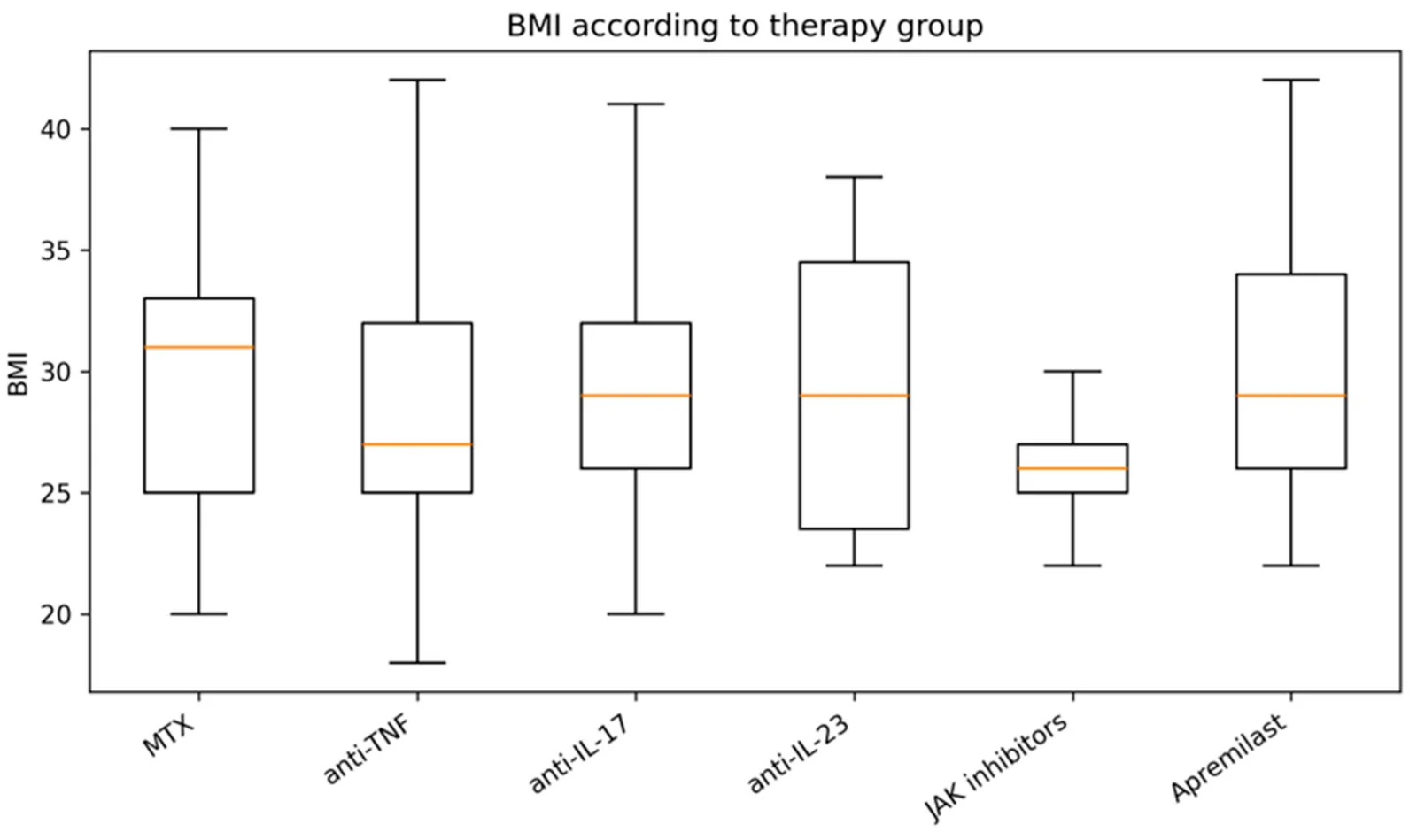
BMI according to therapy group.

Interestingly, no robust therapy-specific separation emerged for several metabolic parameters, including HOMA-IR, HbA1c, triglycerides, and obesity prevalence. Similarly, DAPSA and HAQ distributions did not demonstrate major statistically robust differences between therapeutic groups (DAPSA and HAQ distributions according to treatment group are shown in Figure 11 and Figure 12, respectively, while BMI distribution is reported in Figure 13). These findings suggest that the inflammatory-metabolic phenotype associated with obesity in PsA was observed across patients receiving different therapeutic classes. Metabolic abnormalities were observed in patients receiving biologic or targeted therapies at the time of assessment; however, the cross-sectional design does not allow conclusions regarding their persistence over time or their response to treatment.

### 4.9. Clinical Interpretation

The absence of strong therapy-specific differences for several metabolic variables should be interpreted cautiously. The present dataset reflects a real-world cohort characterized by treatment heterogeneity, variable disease duration, and possible confounding factors related to treatment selection, disease severity, and prior therapeutic exposure. Consequently, the current findings should primarily be considered exploratory and hypothesis-generating.

## 5. Discussion

In the present study, we investigated the immunometabolic landscape of patients with PsA, employing a comprehensive, multidimensional analytical framework. Our findings suggest that obesity is associated with a distinct immunometabolic profile characterized by a heightened inflammatory burden, greater insulin resistance, and a range of adverse metabolic features, which together appear to correlate with diminished functional status.

Multidimensional analyses supported the existence of specific obesity-associated immunometabolic patterns, indicating that this patient subgroup exhibits a complex pathogenic signature. These observations underscore the clinical relevance of recognizing the immunometabolic interplay inherent in PsA, as this multidimensional burden likely shapes the overall disease experience [26].

Our observation that obese patients with PsA exhibited a markedly elevated inflammatory burden is consistent with a growing body of evidence linking increased BMI to heightened systemic inflammation in this population [19,27]. Previous research has frequently identified an association between adiposity and higher levels of acute-phase reactants, such as CRP, suggesting that obesity in PsA may identify a subgroup characterized by a more pronounced inflammatory phenotype [9,19].

This systemic inflammatory amplification is thought to be mediated, at least in part, by the endocrine activity of adipose tissue, which can release various pro-inflammatory cytokines and adipokines that potentially perpetuate chronic immune activation [11,12,28]. While our findings support the perspective that obesity may be associated with an additional inflammatory burden, the underlying interaction between adiposity-induced immune responses and the persistent inflammatory environment in PsA remains complex. This interaction highlights a potential broader immunometabolic dysregulation, pointing toward the relevance of underlying metabolic disturbances that may further modulate this inflammatory state. Building on this understanding, our results further highlight a substantial metabolic burden in patients with PsA. We observed markers of glucose dysregulation, including HOMA-IR and HbA1c, which, together with observed lipid abnormalities, are consistent with previous observations highlighting a heightened risk of metabolic syndrome and insulin resistance in this population [11,12]. These metabolic impairments, often observed in the context of obesity, have been linked to increased cardiometabolic risk in patients with PsA [9,19,29,30,31]. While the exact mechanisms underlying this increased metabolic susceptibility remain under investigation, our results support the growing body of evidence indicating that metabolic abnormalities, including insulin resistance and dyslipidemia, represent a substantial component of the overall disease burden [11]. This association suggests that metabolic and inflammatory pathways are closely interconnected, forming a complex axis that likely contributes to the clinical expression and long-term morbidity associated with PsA.

This immunometabolic crosstalk appears to be orchestrated by adipose tissue, which acts as a metabolically active endocrine organ capable of perpetuating systemic inflammation [11]. The resulting state of “metaflammation”—characterized by adipose tissue dysfunction and immune cell infiltration—may directly exacerbate clinical severity, as pro-inflammatory mediators such as IL-6 and TNF-α facilitate both insulin resistance and enhanced acute-phase responses [11,28,32,33,34]. Furthermore, an altered adipokine profile, involving elevated leptin and resistin alongside diminished adiponectin, potentially functions as a critical bridge that reinforces the interplay between metabolic impairment and chronic inflammatory signaling [9,28].

Beyond these laboratory and metabolic features, our findings underscore the clinical significance of obesity in shaping the broader patient experience of PsA. Our results are consistent with previous literature indicating that increased BMI is associated with heightened patient-reported disease activity, greater functional impairment, and diminished quality of life [19,27]. Specifically, obese individuals in our cohort exhibited elevated scores on assessments of physical disability, such as the HAQ, alongside inferior PRO measures [19,27]. These associations, which have been documented across various cohorts, suggest that obesity may identify patients with a more complex clinical phenotype that transcends standard measures of joint inflammation [9,11]. Consequently, the greater burden of functional impairment observed in this subgroup highlights the need for a comprehensive management strategy that extends beyond disease activity control alone. These considerations are particularly relevant when interpreting the potential influence of different therapeutic approaches on long-term patient outcomes.

This therapeutic stratification also necessitates consideration, as real-world treatment exposure represents a potential source of biological heterogeneity that could modulate both inflammatory and metabolic profiles. Specifically, while a higher BMI has been associated with a less favourable response to both conventional DMARDs and TNF inhibitors [7,10,27], the interplay between metabolic status and treatment-induced modulation of systemic inflammation remains complex. It is important to acknowledge that patients receiving different therapeutic regimens may represent clinically distinct populations, and therefore, variations in inflammatory biomarkers and metabolic parameters across these groups should be interpreted with caution. To reduce the potential impact of measured confounding, we additionally performed multivariable analyses adjusting for age, sex, disease duration, disease activity, hypertension, type 2 diabetes mellitus, and current therapy class. These adjusted analyses confirmed that the principal associations between obesity and the major inflammatory, metabolic, and functional outcomes persisted after accounting for the available covariates. While biologic and targeted synthetic DMARDs have been associated with shifts in systemic cytokine levels and potential improvements in cardiometabolic markers [20,23], observational studies face challenges in distinguishing these treatment-related effects from the patient’s underlying immunometabolic profile. However, differences observed across treatment groups are likely to reflect the clinical characteristics of patients receiving different therapeutic strategies rather than treatment effects per se and should therefore be interpreted with caution.

Ultimately, this complexity underscores the necessity of accounting for therapeutic exposure when evaluating the immunometabolic phenotype of PsA, a perspective that informs our subsequent analysis of the multidimensional burden and systemic interactions.

To further explore the complex interactions underlying this immunometabolic phenotype, we employed multidimensional analyses and machine-learning approaches, which helped provide additional insight into the structured relationships between inflammatory, metabolic, and clinical variables [35,36]. These integrative methods supported the presence of discernible patterns where obesity-associated inflammatory and metabolic abnormalities tend to cluster, highlighting a cohesive immunometabolic profile that is consistent with the findings obtained through our conventional statistical analyses [37,38]. Importantly, these multidimensional approaches complemented, rather than replaced, the conventional multivariable analyses, providing a broader systems-level representation of the immunometabolic phenotype. Rather than evaluating isolated biomarkers, these multidimensional approaches underscore the value of integrating multiple biological domains to better characterize the heterogeneity observed in PsA [37,39]. While these models provided a useful framework for exploring the systemic nature of the disease, it is essential to emphasize that they serve as complementary tools for pattern recognition, and their application should be viewed within the context of the broader, complex clinical landscape. The predictive performance observed in the present study should therefore be interpreted as exploratory rather than definitive. Future studies including larger independent cohorts, external validation, and reporting of confidence intervals for model performance will be required before these approaches can be considered for clinical implementation.

Our findings suggest that obesity may serve as a clinically useful marker for identifying patients with a broader immunometabolic burden [9,11]. Consequently, clinicians may consider integrating comprehensive metabolic and cardiovascular risk assessment into routine rheumatology practice to improve the overall characterization of patients with PsA [9,19]. Such assessments, which monitor cardiometabolic comorbidities alongside inflammatory disease activity, may facilitate a more holistic clinical evaluation [9]. Furthermore, addressing the multifaceted needs of these patients may involve a multidisciplinary framework, incorporating collaborative care across rheumatology, cardiology, endocrinology, and nutrition specialties [20]. Recognizing this immunometabolic phenotype may aid in optimizing clinical management, thereby potentially improving patient-reported outcomes and functional status in individuals with a high comorbidity burden [19,27]. Overall, our findings indicate that obesity is consistently associated with a multidimensional inflammatory, metabolic, and functional profile in PsA, even after adjustment for the principal available clinical and therapeutic covariates. However, longitudinal studies incorporating more comprehensive information on pharmacological exposure and disease evolution will be required to clarify the temporal and mechanistic relationships underlying these associations.

IL-22 may also represent an additional component of the immunometabolic network in PsA. In addition to its involvement in psoriatic inflammation, IL-22 has been implicated in the regulation of lipid metabolism, glucose homeostasis, and adipose tissue function [40,41,42]. It may therefore provide a further biological link between immune activation and metabolic dysfunction. However, IL-22 was not measured in our cohort, and its contribution to the observed immunometabolic phenotype could not be directly assessed.

Our study, while offering relevant insights, should be interpreted in light of several limitations alongside its strengths. A major strength is the real-world cohort design, which enabled a comprehensive assessment of clinical, inflammatory, metabolic, and cytokine-related variables, including insulin resistance through HOMA-IR. The integration of multiple biological domains within a single analytical framework, supported by multidimensional machine-learning techniques, provided a broad characterization of the PsA immunometabolic phenotype. However, the cross-sectional and monocentric design precludes assessment of temporality and causal inference and remains susceptible to residual confounding. Although multivariable analyses accounted for the principal available demographic, clinical, comorbidity-related, inflammatory, metabolic, and current therapeutic factors, residual confounding cannot be excluded. In particular, information on smoking status, physical activity, dietary habits, cardiovascular disease, cumulative corticosteroid exposure, lipid-lowering and glucose-lowering medications, previous PsA treatments, treatment duration, and prior treatment response was not consistently available and therefore could not be incorporated into the adjusted models. Current treatment allocation may also be affected by confounding by indication, since patients receiving different therapies may differ in disease history, severity, and previous treatment response. In addition, BMI represents an indirect surrogate of adiposity, and the limited sample size in some therapeutic subgroups may reduce the precision and generalizability of treatment-stratified estimates. Moreover, the present machine-learning analyses should be considered exploratory because no external validation cohort or nested cross-validation strategy was available. Consequently, the reported predictive performance may overestimate generalizability and requires confirmation in independent multicenter cohorts. Despite these limitations, the study provides a rigorous and integrated description of the relationships among inflammatory, metabolic, functional, and therapeutic variables in PsA. 

## 6. Conclusions

This study underscores that PsA should be recognized as a systemic condition characterized by a complex interplay between chronic inflammation and cardiometabolic dysregulation. The observed coexistence of obesity with systemic inflammation and functional impairment supports the conceptualization of obesity as a critical component of the immunometabolic burden in PsA, rather than a mere comorbidity. These findings support the integration of metabolic and cardiovascular assessment into routine rheumatology practice and highlight obesity as an important marker of increased immunometabolic and clinical burden in PsA. The observed associations may help identify patients requiring closer metabolic and cardiovascular assessment. Consequently, integrating comprehensive metabolic and cardiovascular risk assessments into routine rheumatology practice may assist in identifying individuals who could benefit from more intensive, multidisciplinary management strategies. Such a holistic approach could facilitate improved clinical evaluation and the optimization of care for this multifaceted disease. Future research is needed to further characterize the mechanisms underpinning this obesity-associated immunometabolic phenotype and its longitudinal impact on patient outcomes.

## Figures and Tables

**Table 1:** Baseline characteristics of the study population according to obesity status.

Variable	Total Cohort (*n* = 224)	Non-Obese (*n* = 130)	Obese (*n* = 94)	*p* Value
Age, years	62.0 [56.0–71.0]	60.5 [55.0–70.0]	63.0 [56.0–72.0]	0.314
Female sex, *n* (%)	132 (58.9%)	75 (57.7%)	57 (60.6%)	0.665
Disease duration, months	36.0 [10.0–70.0]	36.0 [10.0–60.0]	36.0 [19.5–70.0]	0.582
BMI, kg/m^2^	28.0 [25.0–32.0]	25.5 [24.0–27.0]	33.0 [31.0–35.0]	<0.001
DAPSA	4.0 [3.0–5.0]	4.0 [3.0–5.0]	4.1 [3.2–5.0]	0.966
HAQ	0.50 [0.25–0.75]	0.50 [0.25–0.50]	0.50 [0.50–0.75]	<0.001
ESR, mm/h	13.0 [8.0–21.0]	10.0 [7.0–18.0]	15.0 [9.0–22.8]	0.006
CRP, mg/dL	0.24 [0.08–0.51]	0.13 [0.06–0.33]	0.31 [0.19–0.58]	<0.001
SAA, mg/L	6.4 [6.4–10.6]	6.4 [6.4–10.2]	7.7 [6.4–12.7]	0.008
IL-6, pg/mL	2.8 [1.5–4.3]	2.4 [1.3–3.6]	3.4 [2.0–4.5]	<0.001
Insulin, μU/mL	7.1 [5.2–10.2]	6.1 [4.1–7.6]	8.0 [6.2–12.1]	<0.001
HbA1c, mmol/mol	37.0 [35.0–40.3]	37.0 [34.0–38.0]	38.0 [35.3–42.5]	0.003
HOMA-IR	1.26 [0.79–2.90]	1.21 [0.79–1.94]	1.29 [0.91–3.50]	0.007
HDL-C, mg/dL	66.5 [54.0–74.0]	69.0 [55.3–79.0]	64.0 [54.0–73.0]	0.004
Triglycerides, mg/dL	89.0 [67.8–120.5]	85.0 [64.8–112.8]	106.0 [72.0–131.8]	0.019
Hypertension, *n* (%)	26 (11.6%)	9 (6.9%)	17 (18.1%)	0.0117
Hypercholesterolemia, *n* (%)	32 (14.3%)	15 (11.5%)	17 (18.1%)	0.1802
Type 2 diabetes mellitus, *n* (%)	20 (8.9%)	8 (6.2%)	12 (12.8%)	0.1000

Abbreviations: BMI, body mass index; DAPSA, Disease Activity Index for Psoriatic Arthritis; HAQ, Health Assessment Questionnaire; ESR, erythrocyte sedimentation rate; CRP, C-reactive protein; SAA, serum amyloid A; IL-6, interleukin-6; HbA1c, glycated hemoglobin; HOMA-IR, Homeostasis Model Assessment of Insulin Resistance; HDL-C, high-density lipoprotein cholesterol.

**Table 2:** Spearman Correlations Between BMI and Clinical, Inflammatory, and Metabolic Variables.

Variable	Spearman’s ρ	*p* Value	FDR-Adjusted *p* Value
C-Reactive Protein (CRP)	0.383	<0.0001	<0.0001
Health Assessment Questionnaire (HAQ) Score	0.351	<0.0001	<0.0001
Interleukin-6 (IL-6)	0.295	<0.0001	0.0001
Insulin	0.289	<0.0001	0.0001
Erythrocyte Sedimentation Rate (ESR)	0.245	0.0002	0.0009
Serum Amyloid A (SAA)	0.228	0.0006	0.0020
Glycated Hemoglobin (HbA1c)	0.199	0.0028	0.0084
High-Density Lipoprotein Cholesterol (HDL-C)	−0.189	0.0045	0.0119
Disease Activity Index for Psoriatic Arthritis (DAPSA)	0.183	0.0060	0.0140
Homeostasis Model Assessment of Insulin Resistance HOMA-IR	0.154	0.0213	0.0232

Abbreviations: BMI, body mass index; CRP, C-reactive protein; HAQ, Health Assessment Questionnaire; IL-6, interleukin-6; ESR, erythrocyte sedimentation rate; SAA, serum amyloid A; HbA1c, glycated hemoglobin; HDL-C, high-density lipoprotein cholesterol; DAPSA, Disease Activity Index for Psoriatic Arthritis; FDR, false discovery rate.

**Table 3:** Adjusted associations between obesity and the principal inflammatory, metabolic, and functional outcomes.

Outcome	Statistical Model	Adjusted Estimate	95% CI	*p* Value	FDR-Adjusted *p* Value
CRP	Robust linear regression, log-transformed outcome	Ratio 2.10	1.55–2.85	<0.0001	<0.0001
Fasting insulin	Robust linear regression, log-transformed outcome	Ratio 1.32	1.11–1.56	0.0018	0.0018
HAQ category	Ordinal logistic regression	Proportional OR 3.10	1.79–5.37	<0.0001	<0.0001

**Table 4:** Comorbidity distribution according to obesity status.

Comorbidity	Non-Obese %	Obese %	Odds Ratio	*p* Value	Interpretation
Hypercholesterolemia	11.5	18.1	1.69	0.1802	Higher in obesity
Hypertension	6.9	18.1	2.97	0.0117	Higher in obesity
Non-insulin-dependent diabetes mellitus (NIDDM)	6.2	12.8	2.23	0.1000	Higher in obesity

**Table 5:** Performance of the machine-learning models for obesity prediction.

Model	Mean CV AUC	Accuracy	Balanced Accuracy	Sensitivity	Precision
Random Forest	0.733	0.692	0.685	0.638	0.632
Gradient Boosting	0.699	0.621	0.604	0.500	0.553
Logistic Regression	0.637	0.625	0.614	0.543	0.554

Abbreviations: AUC, area under the receiver operating characteristic curve; CV, cross-validation. Values represent the mean performance across the five cross-validation folds. Mean CV AUC indicates the average area under the ROC curve across the validation folds.

**Table 6:** Top Random Forest features associated with obesity.

Feature	Importance
CRP	0.1342
Insulin	0.1108
IL-6	0.0624
HAQ	0.0586
HOMA-IR	0.0558
ESR	0.0548
HbA1c	0.0505
DAPSA	0.0480
TG	0.0449
HDL	0.0408

Abbreviations: CRP, C-reactive protein; IL-6, interleukin-6; HAQ, Health Assessment Questionnaire; HOMA-IR, Homeostasis Model Assessment of Insulin Resistance; ESR, erythrocyte sedimentation rate; HbA1c, glycated hemoglobin; DAPSA, Disease Activity Index for Psoriatic Arthritis; TG, triglycerides; HDL, high-density lipoprotein cholesterol.

**Table 7:** Principal component (PC) explained variance.

Component	Explained Variance	Cumulative Variance
PC1	13.1%	13.1%
PC2	10.7%	23.8%
PC3	9.4%	33.2%

**Table 8:** Demographic and Disease Characteristics of the Study Population (*n* = 224). Continuous variables are presented as mean ± standard deviation (SD), median (interquartile range, IQR), and range.

Variable	Mean ± SD	Median (IQR)	Range
Age (years)	62.33 ± 11.20	62.0 (56.0–71.0)	30–85
Disease duration (months)	50.46 ± 43.54	36.0 (10.0–70.0)	1–240
DAPSA score	4.17 ± 2.37	4.00 (3.02–5.00)	0.10–28.09

Abbreviations: DAPSA, Disease Activity in Psoriatic Arthritis; IQR, interquartile range; SD, standard deviation.

## Data Availability

The raw data supporting the conclusions of this article will be available from the authors without undue reservation.

## Supplementary Materials

The following supporting information can be downloaded at: https://www.mdpi.com/article/10.3390/diseases14080296/s1, Table S1: Inflammatory and metabolic parameters according to current therapeutic regimen in patients with psoriatic arthritis; Table S2: Comparison of all measured laboratory variables according to obesity status.
