# Supplementary material for: Body Mass Index Is Associated with the Immunometabolic Profile in Psoriatic Arthritis: Real-Life Data
Source: Diseases. 2026 Aug 17;14(8):296. doi: 10.3390/diseases14080296 (PMC13512439; doi:10.3390/diseases14080296)
Supplement: Supplementary file 1 [file diseases-14-00296-s001.zip › Supplementary Table S1.pdf]

Supplementary Table S1. Inflammatory and metabolic parameters according to current therapeutic regimen in patients with psoriatic arthritis.

Distribution of inflammatory and metabolic biomarkers according to current therapeutic regimen in patients with psoriatic arthritis. Data are presented as median (interquartile range, IQR). Comparisons among treatment groups were performed using the Kruskal–Wallis test. Owing to the observational cross-sectional design, the limited sample size of several therapeutic subgroups, and the potential for confounding by indication, these analyses should be considered exploratory and hypothesis-generating. Abbreviations: CRP, C-reactive protein; ESR, erythrocyte sedimentation rate; SAA, serum amyloid A; IL-6, interleukin-6; HbA1c, glycated hemoglobin; HOMA-IR, homeostasis model assessment of insulin resistance; TG, triglycerides.

**Therapy**	**n**	**CRP**	**ESR**	**SAA**	**IL-6**	**Insulina**	**HOMA-IR**	**Hb glicata**	**TG**
Etanercept	54	0.17 (0.06–0.39)	11.00 (7.00–18.00)	6.40 (6.40–8.20)	2.10 (1.20–3.40)	7.60 (5.45–10.80)	1.26 (0.79–3.34)	37.00 (35.00–42.50)	83.50 (69.00–113.50)
Adalimumab	37	0.20 (0.05–0.30)	9.00 (6.00–14.00)	6.40 (6.40–6.40)	2.80 (1.60–3.40)	6.60 (5.10–9.00)	1.26 (0.79–2.34)	37.00 (34.00–40.00)	108.00 (67.00–133.00)
Secukinumab	33	0.24 (0.09–0.42)	15.00 (10.00–19.00)	7.40 (6.40–14.00)	2.70 (1.90–3.60)	6.90 (5.20–7.60)	1.26 (0.79–3.00)	37.00 (35.00–38.00)	95.00 (59.00–118.00)
MTX	32	0.17 (0.10–0.39)	15.00 (8.00–22.00)	8.40 (6.40–12.72)	2.90 (1.88–3.52)	7.30 (5.60–12.70)	1.26 (0.79–4.11)	38.00 (35.00–41.75)	87.50 (70.75–115.25)
Ixekizumab	17	0.30 (0.24–0.39)	11.00 (7.00–21.00)	6.40 (6.40–10.40)	3.30 (2.50–4.60)	7.00 (5.40–8.30)	1.26 (0.79–1.78)	37.00 (34.00–39.00)	88.00 (67.00–129.00)
Bimekizumab	12	0.64 (0.20–1.71)	22.00 (10.25–25.75)	15.30 (7.60–19.65)	3.85 (2.17–7.40)	6.35 (4.90–8.68)	1.22 (0.79–3.12)	37.00 (34.75–39.25)	107.00 (93.00–109.25)
No recorded treatment	9	0.18 (0.10–0.30)	9.00 (7.00–14.00)	6.40 (6.40–9.10)	2.30 (1.50–5.10)	5.80 (3.10–7.60)	1.26 (0.64–1.49)	37.00 (34.00–38.00)	112.00 (85.00–112.00)

Apremilast	7	0.80 (0.53–2.00)	28.00 (12.50–51.50)	14.60 (12.60–47.05)	4.90 (3.45–12.35)	7.60 (3.90–9.50)	0.91 (0.71–1.22)	38.00 (37.00–38.00)	78.00 (68.00–86.00)
Certolizumb	5	0.24 (0.08–0.36)	13.00 (10.00–21.00)	6.40 (6.40–6.40)	3.40 (2.00–3.60)	12.30 (4.10–13.00)	1.21 (0.79–3.75)	41.00 (38.00–46.00)	123.00 (95.00–197.00)
Guselkumab	4	0.29 (0.25–0.54)	17.00 (9.50–24.50)	13.85 (11.20–15.60)	1.65 (1.20–2.58)	11.10 (7.30–34.20)	1.26 (1.02–3.23)	39.00 (30.25–42.00)	71.00 (56.75–83.75)
Tofacitinib	3	0.39 (0.21–0.65)	13.00 (7.50–37.00)	6.40 (6.40–9.45)	4.30 (2.75–5.05)	7.60 (5.70–7.95)	1.26 (1.02–1.58)	38.00 (36.50–38.00)	78.00 (71.00–150.00)
Upadacitinib	3	0.02 (0.02–0.70)	10.00 (9.50–21.50)	6.40 (6.40–22.50)	0.60 (0.35–5.00)	11.10 (7.60–12.60)	2.00 (1.60–2.88)	46.00 (40.50–47.00)	129.00 (86.50–175.50)
Infliximab	3	0.12 (0.08–0.34)	16.00 (9.50–17.00)	6.40 (6.40–9.35)	2.60 (1.35–3.00)	3.80 (3.80–4.70)	0.79 (0.79–0.98)	35.00 (35.00–38.00)	78.00 (72.50–137.50)
guselkumab	2	0.66 (0.64–0.68)	37.50 (22.75–52.25)	17.05 (12.07–22.02)	7.95 (7.47–8.43)	17.70 (12.70–22.70)	2.30 (2.05–2.55)	40.00 (39.50–40.50)	264.00 (198.00–330.00)
mtx	1	0.02 (0.02–0.02)	4.00 (4.00–4.00)	6.40 (6.40–6.40)	2.10 (2.10–2.10)	7.60 (7.60–7.60)	1.26 (1.26–1.26)	38.00 (38.00–38.00)	47.00 (47.00–47.00)
Ustekinumab	1	0.59 (0.59–0.59)	9.00 (9.00–9.00)	6.40 (6.40–6.40)	6.70 (6.70–6.70)	2.80 (2.80–2.80)	0.66 (0.66–0.66)	34.00 (34.00–34.00)	129.00 (129.00–129.00)
leflunomide	1	0.03 (0.03–0.03)	21.00 (21.00–21.00)	23.80 (23.80–23.80)	3.00 (3.00–3.00)	9.50 (9.50–9.50)	1.26 (1.26–1.26)	35.00 (35.00–35.00)	91.00 (91.00–91.00)
