# Supplementary material for: Body Mass Index Is Associated with the Immunometabolic Profile in Psoriatic Arthritis: Real-Life Data
Source: Diseases. 2026 Aug 17;14(8):296. doi: 10.3390/diseases14080296 (PMC13512439; doi:10.3390/diseases14080296)
Supplement: Supplementary file 1 [file diseases-14-00296-s001.zip › Supplementary Table S2.pdf]

Supplementary Table S2. Comparison of all measured laboratory variables according to obesity status

Laboratory parameter	BMI <30 (n = 130) Median (IQR)	BMI ≥30 (n = 94) Median (IQR)	Raw p value	FDR-adjusted p value
ESR (mm/h)	10.0 (7.0–18.0)	15.0 (9.0–22.8)	0.006	0.022
CRP (mg/dL)	0.1 (0.1–0.3)	0.3 (0.2–0.6)	<0.001	<0.001
MRP	1.7 (0.8–1.7)	1.7 (1.3–1.8)	0.069	0.130
SAA (mg/L)	6.4 (6.4–10.2)	7.7 (6.4–12.7)	0.008	0.022
TNF (pg/mL)	0.1 (0.1–78.0)	0.1 (0.1–3.6)	0.818	0.869
IL-6 (pg/mL)	2.4 (1.3–3.6)	3.3 (2.0–4.5)	0.026	0.056
IL-10 (pg/mL)	0.2 (0.1–0.8)	0.2 (0.1–1.0)	0.896	0.896
IL-17 (pg/mL)	0.1 (0.1–0.3)	0.1 (0.1–0.1)	0.098	0.152
IL-8 (pg/mL)	0.1 (0.1–1.0)	0.1 (0.1–3.4)	0.232	0.304
Fasting glucose (mg/dL)	90.0 (81.0–101.0)	93.0 (85.0–101.2)	0.135	0.191
Fasting insulin (µU/mL)	6.0 (4.1–7.6)	7.9 (6.2–12.1)	<0.001	<0.001
HbA1c (mmol/mol)	37.0 (34.0–38.0)	38.0 (35.2–42.5)	0.003	0.015
HOMA-IR	1.2 (0.8–1.9)	1.3 (0.9–3.3)	0.007	0.022
Total cholesterol (mg/dL)	192.0 (164.0–228.5)	195.5 (173.0–228.8)	0.737	0.836
LDL-C (mg/dL)	118.0 (90.2–138.8)	120.0 (96.0–137.8)	0.582	0.706
HDL-C (mg/dL)	69.0 (55.2–79.0)	64.0 (54.0–73.0)	0.083	0.140
Triglycerides (mg/dL)	85.0 (64.8–112.8)	106.0 (72.0–131.8)	0.019	0.046

Data are presented as median (interquartile range). Comparisons between obese and non-obese patients were performed using the Mann–Whitney U test. Raw p values were adjusted using the Benjamini–Hochberg false discovery rate procedure. Four implausible HOMA-IR values identified during data quality control were recalculated using the standard HOMA-IR formula before statistical analysis.
